# Anodal transcranial direct current stimulation boosts synaptic plasticity and memory in mice via epigenetic regulation of Bdnf expression

**DOI:** 10.1038/srep22180

**Published:** 2016-02-24

**Authors:** Maria Vittoria Podda, Sara Cocco, Alessia Mastrodonato, Salvatore Fusco, Lucia Leone, Saviana Antonella Barbati, Claudia Colussi, Cristian Ripoli, Claudio Grassi

**Affiliations:** 1 Institute of Human Physiology, Università Cattolica, Largo F. Vito 1, 00168 Rome, Italy; 2 Institute of Cell Biology and Neurobiology, CNR, Monterotondo, Italy; 3 San Raffaele Pisana Scientific Institute for Research, Hospitalization and Health Care, 00163 Rome, Italy

## Abstract

The effects of transcranial direct current stimulation (tDCS) on brain functions and the underlying molecular mechanisms are yet largely unknown. Here we report that mice subjected to 20-min anodal tDCS exhibited one-week lasting increases in hippocampal LTP, learning and memory. These effects were associated with enhanced: i) acetylation of brain-derived neurotrophic factor (Bdnf) promoter I; ii) expression of *Bdnf* exons I and IX; iii) Bdnf protein levels. The hippocampi of stimulated mice also exhibited enhanced CREB phosphorylation, pCREB binding to *Bdnf* promoter I and recruitment of CBP on the same regulatory sequence. Inhibition of acetylation and blockade of TrkB receptors hindered tDCS effects at molecular, electrophysiological and behavioral levels. Collectively, our findings suggest that anodal tDCS increases hippocampal LTP and memory via chromatin remodeling of *Bdnf* regulatory sequences leading to increased expression of this gene, and support the therapeutic potential of tDCS for brain diseases associated with impaired neuroplasticity.

Numerous studies provided molecular and functional evidence linking synaptic plasticity in certain brain areas to specific motor and cognitive functions^1^,^2^. Additionally, altered synaptic plasticity has been associated to neuropsychiatric disorders^3^ and cognitive impairment^4^. Thus targeting synaptic plasticity may provide a major breakthrough for therapeutic interventions.

A very promising approach is the use of non-invasive brain stimulation techniques and, in particular, transcranial direct current stimulation (tDCS) due to its ease of use and beneficial effects on brain functions in healthy subjects as well as in patients with neurological and psychiatric diseases^5^,^6^,^7^,^8^,^9^.

Early studies demonstrated that tDCS induces polarity-dependent changes in membrane excitability, with anodal tDCS causing depolarization and cathodal tDCS hyperpolarization of membrane potential in neurons of the stimulated areas^10^,^11^,^12^,^13^.

Changes in neuronal excitability and behaviors were reported to persist after tDCS^14^,^15^,^16^ thus raising the question on whether synaptic plasticity phenomena are involved in tDCS’s action^17^. So far, a direct link between tDCS and synaptic plasticity was proposed by very few studies, including ours. Fritsch *et al.*^18^ showed that anodal DCS applied to mouse motor cortex *in vitro* induced NMDA receptor (NMDAR)-dependent long-term potentiation (LTP) when the stimulus was given concomitantly with synaptic activation. We demonstrated that anodal stimulation applied to hippocampal slices increased the magnitude of LTP at CA3-CA1 synapses whereas cathodal DCS decreased it^19^. More recently, Rohan *et al.*^20^ reported that *in vivo* tDCS increased LTP and paired-pulse facilitation in rat hippocampus.

To advance our understanding of tDCS action on brain functions we studied the effects and the duration of anodal tDCS on hippocampal synaptic plasticity at electrophysiological, molecular and behavioral levels. We focused our attention on molecular mechanisms potentially responsible for long-lasting effects. Bdnf was considered a good candidate given its involvement in LTP and memory^21^,^22^. The structure of *Bdnf*, i.e., eight 5′ non-coding exons and a 3′ coding exon (IX) under the control of multiple promoters, allows differential regulation of gene expression by several stimuli^23^,^24^. In particular, memory formation is accompanied by changes in chromatin structure involving enhanced histone 3 (H3) acetylation on specific *Bdnf* promoters, leading to increased expression of exon I and IX mRNAs^25^. These data grounded the rationale to test whether anodal tDCS affected *Bdnf* expression via epigenetic modifications, in a fashion resembling neuronal activation.

## Results

### Long-term potentiation is enhanced in mouse hippocampus following anodal tDCS

We previously demonstrated that *in vitro* DCS of hippocampal slices induced polarity-dependent modulation of LTP at CA3-CA1 synapses^19^. As cornerstone data for further analysis we first ascertained whether tDCS (350 μA) applied for 20 min to the skull of behaving mice and targeting the hippocampal formation elicited similar effects on hippocampal LTP.

**Figure 1:**
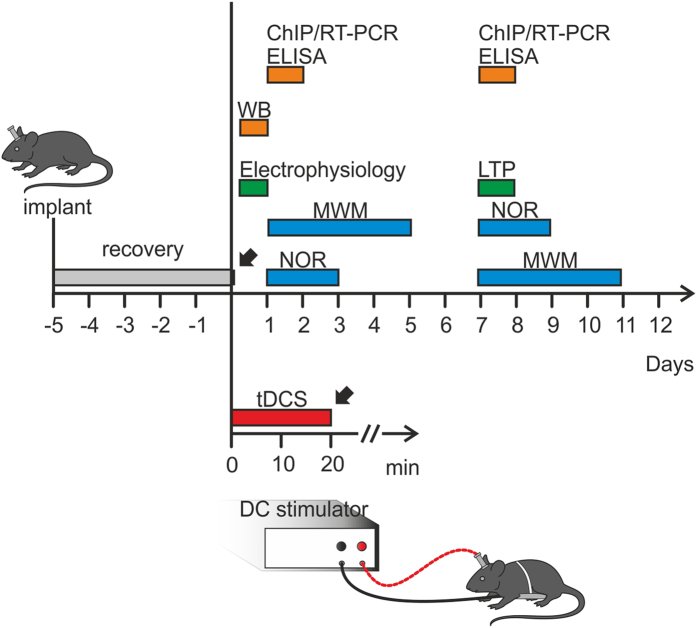
Schematic representation of the experimental design and time schedule of the protocols. Adult male C57bl/6 mice were implanted by an epicranial electrode and were allowed to recover from surgery before undergoing tDCS (350 μA for 20 min) or sham stimulation. Different groups of animals were used for each experimental protocol at different time points. ChIP: Chromatin Immunoprecipitation; Electrophysiology (i.e., LTP recordings, I/O relationship, paired-pulse ratio, AMPA/NMDA ratio); ELISA: Enzyme-Linked Immunosorbent Assay; LTP (i.e, LTP field recordings); MWM: Morris Water Maze test; NOR: Novel Object Recognition test; RT-PCR: semiquantitative Reverse Transcription-PCR or quantitative Real-Time PCR; WB: Western immunoblot analysis.

Field evoked post-synaptic potentials (fEPSPs) were measured in the CA1 area after standard high-frequency stimulation (HFS) of Schaffer collaterals and LTP was measured in slices from both sham- (control) and tDCS-stimulated mice that were sacrificed soon after the tDCS (Fig. 1).

**Figure 2:**
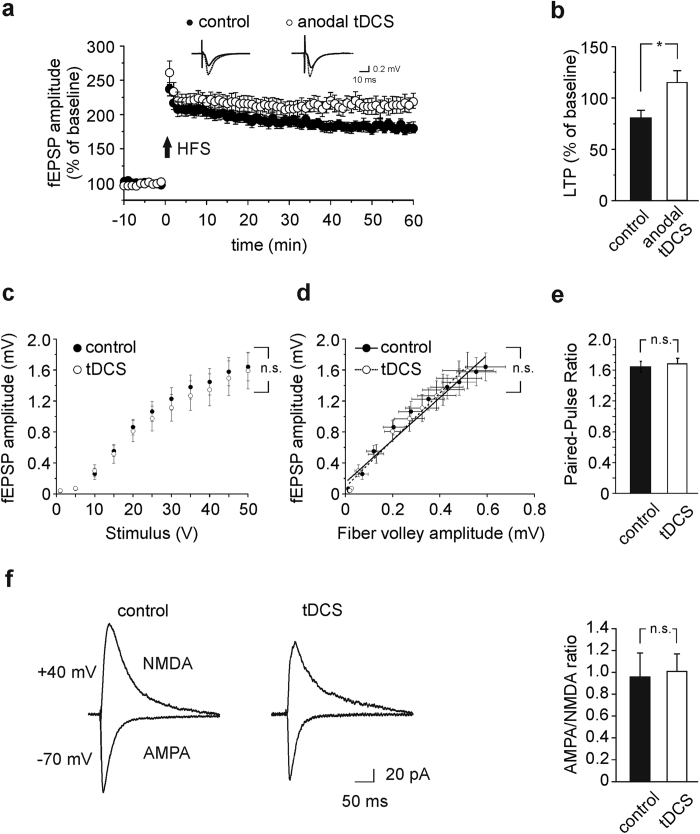
LTP at CA3-CA1 synapses is enhanced in slices from mice subjected to anodal tDCS with no changes in basal synaptic transmission. (**a**) Time course of LTP at CA3-CA1 synapses induced by HFS (4 trains of 50 stimuli at 100 Hz for 500 ms repeated every 20 seconds) delivered at time 0 (arrow) in hippocampal slices obtained from sham-stimulated controls (n = 16 slices from 8 mice) and mice subjected to anodal tDCS (n = 10 slices from 5 mice). Results are expressed as percentages of baseline fEPSP amplitude ( = 100%). Insets show representative fEPSPs at baseline (solid line) and during the last 5 min of LTP recording (dotted line). Traces are averages of 5 consecutive responses at the time points selected (see Supplementary Table 1 for statistical comparison of normalized fEPSP amplitudes 5 min before and 55–60 min after HFS). (**b**) Bar graphs comparing LTP observed during the last 5 min in control and anodal tDCS-mice (P = 0.01, unpaired Student’s *t*-test). (**c**) fEPSP amplitudes following stimulation of CA3 fibers at increasing intensities are shown for slices obtained from tDCS-mice or controls (same slices used for LTP; P > 0.5 at all stimulus intensities, two-way RM ANOVA, Bonferroni *post-hoc*). (**d**) Input-output curves established by plotting the amplitudes of fEPSPs against the amplitudes of presynaptic fiber volleys at increasing stimulus intensities in hippocampal slices of control and tDCS-mice (same slices used for LTP; P = 0.86, unpaired Student’s *t*-test). (**e**) Responses to paired-pulse stimulation (50 ms inter-stimulus interval) in slices from control and tDCS-mice (same slices used for LTP; P = 0.72, unpaired Student’s *t*-test). (**f**) Representative AMPAR- and NMDAR-mediated EPSCs recorded in CA1 neurons in slices obtained from control and tDCS-mice, and bar graph showing AMPA/NMDA ratio in CA1 pyramidal neurons (n = 14 cells from 3 tDCS-mice *vs.* n = 12 cells from 3 control mice; P = 0.13, Mann-Whitney test). Overall slices from control and tDCS-mice did not show significant differences with regard to parameters shown in (**c–f** ). Data are expressed as mean ± s.e.m; *P < 0.05; n.s., not significant *vs.* control.

Sixty min after HFS slices from mice subjected to anodal tDCS showed significantly greater LTP than controls (115.1 ± 11.8% [n = 10 slices from 5 mice] *vs.* 80.8 ± 7.5% [n = 16 slices from 8 mice]; P = 0.01, unpaired Student’s *t*-test; Fig. 2a,b). Reduced LTP was instead observed in slices from mice undergone to cathodal tDCS (32.8 ± 9.3% [n = 10 slices from 4 mice] *vs.* 88.6 ± 17.5% [n = 9 slices from 4 control mice]; P = 0.004, unpaired Student’s *t*-test; Supplementary Fig. 1a,b). These results indicate that hippocampal synaptic plasticity can be targeted and effectively modulated by *in vivo* tDCS.

A set of experiments was also performed to check whether unilateral tDCS also affected LTP in the contralateral hippocampus. Results showed that LTP values in the right hippocampus were slightly smaller but not significantly different from those obtained in the left side (P = 0.83, unpaired Student’s *t*-test), thus suggesting that tDCS also affected the hippocampus contralateral to the stimulating electrode.

Control experiments were performed to assess the specificity of the observed effects. To this aim we recorded LTP at CA3-CA1 synapses in slices obtained from mice that had undergone sham stimulation (n = 8 slices from 2 mice) or tDCS (n = 6 slices from 2 mice) of the left primary motor cortex^26^. Under these experimental conditions no changes in hippocampal LTP were observed following tDCS (P = 0.59 *vs.* control, unpaired Student’s *t*-test; Supplementary Fig. 1c). Further control experiments were performed to assess tissue viability following tDCS. Histological evaluation by hematoxylin-eosin staining was carried out in 6 mice (n = 3 control and n = 3 receiving anodal tDCS; Supplementary Fig. 1d) and it revealed no signs of neurotrauma^27^.

Our subsequent studies focused on anodal tDCS effects given their potential use to counteract impaired synaptic plasticity in normal aging or disease. Thereinafter animals receiving anodal tDCS are referred to as “tDCS-mice”.

To check whether enhanced LTP following anodal tDCS was due to changes in basal synaptic transmission, we first examined input-output (I/O) curves obtained by plotting fEPSP amplitude against stimulus intensities. No significant differences were found between I/O curves of control and tDCS-mice (same slices used for LTP; P > 0.5 at all stimulus intensities, two-way repeated measure [RM] ANOVA followed by Bonferroni *post-hoc* test; Fig. 2c). Similar results were obtained by plotting the amplitude of presynaptic fiber volleys against the amplitude of fEPSPs (P = 0.86, unpaired Student’s *t*-test; Fig. 2d).

Next, we studied the paired-pulse ratio (PPR) that provides information on neurotransmitter release probability^28^. Stimulation of the Schaffer collateral consistently produced paired-pulse facilitation in all slices tested and the PPR was not significantly different between the two groups (PPR: 1.68 ± 0.07 in slices from tDCS-mice *vs*. 1.64 ± 0.07 in controls; P = 0.72, unpaired Student’s *t*-test; Fig. 2e). Finally, in whole-cell patch-clamp recordings we measured the ratio of AMPAR-mediated evoked post-synaptic currents (EPSCs) to NMDAR-mediated EPSCs, that is a standard test to detect changes in synaptic strength^29^,^30^. This parameter was not significantly affected by tDCS (1.0 ± 0.2 [n = 14 cells from 3 tDCS-mice] *vs.* 0.9 ± 0.3 [n = 12 cells from 3 control mice]; P = 0.13, Mann-Whitney test; Fig. 2f).

Collectively, these data indicate that effects of anodal tDCS on LTP are independent of changes in basal synaptic efficiency.

### Spatial learning and memory are improved in mice subjected to anodal tDCS

Having shown that anodal tDCS increased hippocampal LTP, we determined whether hippocampal-dependent learning and memory were similarly affected by comparing the performance of control and tDCS-mice in the MWM and NOR tasks. These behavioral tests were started 24 h after the end of tDCS or sham stimulation (Fig. 1).

**Figure 3:**
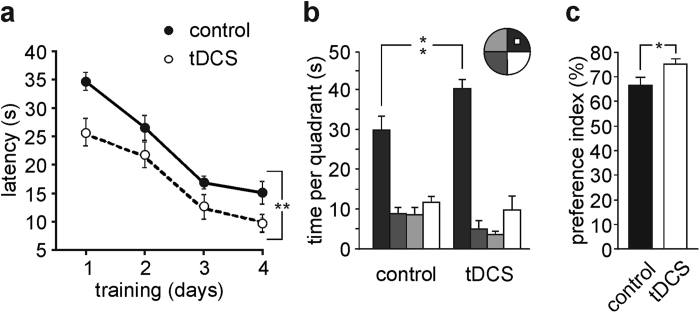
Anodal tDCS improves learning and memory in the MWM and NOR tests started 24 h after stimulation. (**a**) Escape latency to reach the hidden platform of control and anodal tDCS-mice (n = 9 for each group; F_1_,_48_ = 21.2, P < 0.001 for overall acquisition; main effect of days: F_3,48_ = 37.8, P < 0.001, two-way RM ANOVA). (**b**) Time spent in the four quadrants during probe test performed on day 5 of MWM test. In particular, tDCS-mice displayed a significant increase in the time spent in the target quadrant (black bars) compared to control animals (40.6 ± 3.0 s *vs.* 30.3 ± 3.3 s, P < 0.001; F_3,48_ = 4.7, P = 0.006 for interaction between tDCS and time in quadrants; two-way RM ANOVA, Bonferroni *post-hoc*). (**c**) Preference toward the novel object in NOR paradigm (n = 9 for each group; P = 0.04, unpaired Student’s *t*-test). Data are expressed as mean ± s.e.m. *P < 0.05; **P < 0.001.

In the acquisition session of the MWM all mice successfully acquired the task with latency to reach the platform decreasing progressively across training days (main effect of days: F_3_,_48_ = 37.8, P < 0.001, two-way RM ANOVA; Fig. 3a). Notably, tDCS-mice performed better than controls (n = 9 mice/group; F_1_,_48_ = 21.2, P < 0.001 for overall acquisition). As far as the differences we observed on day 1 (main effect of tDCS: F_1,80_ = 10.5, P = 0.005, two-way RM ANOVA), there were no significant differences between control and tDCS-mice in the first two trials (P = 0.29 and P = 0.28 for trial 1 and trial 2, respectively, two-way RM ANOVA, Bonferroni *post-hoc*) indicating equal baselines. Similar results were obtained analyzing the length of the swim path (main effect of days: F_3,48_ = 31.7, P < 0.001; main effect of treatment: F_1,48_ = 14.1, P = 0.002; day 1: P = 0.67 and P = 0.41 for trial 1 and trial 2, respectively, two-way RM ANOVA, Bonferroni *post-hoc*; Supplementary Fig. 2).

In the probe test performed 24 h after the last training trial all mice showed a preference for the target quadrant (main effect of quadrant: F_3,48_ = 71.8, P < 0.001, two-way RM ANOVA). Notably, tDCS-mice spent more time in the target quadrant compared to controls (40.6 ± 3.0 s *vs.* 30.3 ± 3.3 s, P < 0.001; F_3,48_ = 4.7, P = 0.006 for interaction between tDCS and time in quadrants; two-way RM ANOVA, Bonferroni *post-hoc*; Fig. 3b), thus indicating improved spatial memory.

Furthermore, in the NOR tDCS-mice showed a greater preference toward the novel object than control mice (preference index: 75.1 ± 2.3% and 66.5 ± 3.4%, respectively; n = 9 for each group, P = 0.04, unpaired Student’s *t*-test; Fig. 3c), supporting the beneficial effects of anodal tDCS on learning and memory.

### Effects of anodal tDCS on hippocampal LTP, learning and memory persist one week after stimulation

**Figure 4:**
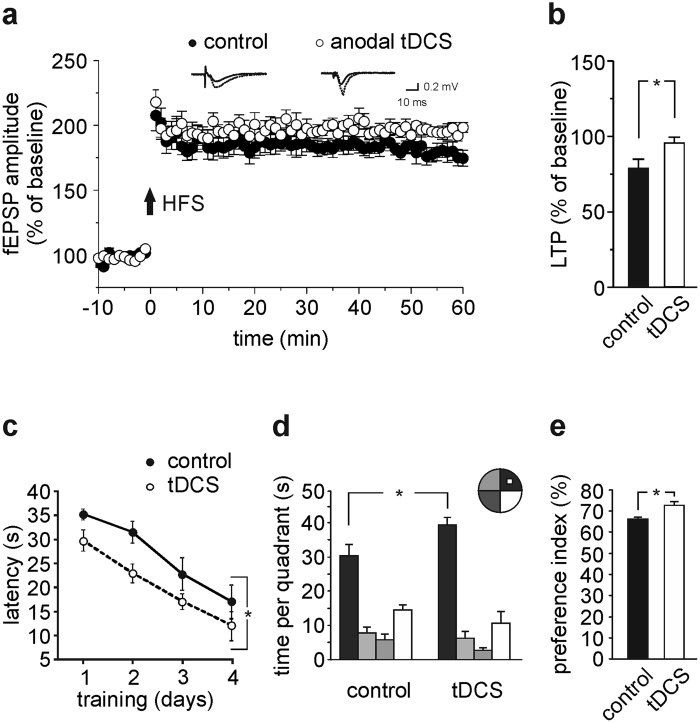
Anodal tDCS-induced enhancement of hippocampal plasticity persists 1 week after stimulation. (**a**) Time course and (**b**) magnitude of LTP induced by HFS protocol in hippocampal slices from mice sacrificed 1 week after tDCS (n = 11 slices from 6 mice) or sham stimulation (n = 9 slices from 5 mice); P = 0.02; unpaired Student’s *t*-test. (**c,d**) Results from MWM test started 1 week after stimulation (n = 9 controls and n = 7 tDCS-mice). (**c**) Escape latency to reach the hidden platform (main effect of days: F_3,42_ = 28.5, P < 0.001, two-way RM ANOVA). tDCS-mice exhibited reduced latency to reach the hidden platform compared to controls (F_1,42_ = 6.8, P = 0.021 for overall acquisition, two-way RM ANOVA). (**d**) Time spent in the four quadrants during probe test (P = 0.0064, two-way RM ANOVA, Bonferroni *post-hoc*). (**e**) Preference toward the novel object in NOR paradigm (n = 8 for each group; P = 0.004, unpaired Student’s *t*-test). Data are expressed as mean ± s.e.m. *P < 0.05.

To gain information on the duration of anodal tDCS effects on hippocampal plasticity we repeated LTP experiments and behavioral tests 1 week after tDCS (Fig. 1). Interestingly, LTP elicited by HFS in slices from tDCS-mice was significantly higher compared to that of control slices (95.5 ± 3.8% [n = 11 slices from 6 tDCS-mice] *vs.* 78.6 ± 6.1% [n = 9 slices from 5 control mice], P = 0.02, unpaired Student’s *t*-test; Fig. 4a,b).

Consistent with the view that hippocampal-dependent learning and memory rely on LTP^31^,^32^, tDCS-mice also performed better than controls in MWM and NOR tests started 1 week after tDCS (Fig. 4c–e). In the MWM all mice displayed decreases in the latency to reach the hidden platform over training days (main effect of days: F_3,42_ = 28.5, P < 0.001, two-way RM ANOVA), however tDCS-mice showed reduced latency to reach the hidden platform than controls (n = 9 control mice and n = 7 tDCS-mice; F_1,42_ = 6.8, P = 0.021 for overall acquisition; Fig. 4c).

The persistence of effect of tDCS on spatial memory was then confirmed by enhanced time spent in the target quadrant by tDCS-mice compared to controls (39.7 ± 2.3 s *vs.* 30.7 ± 3.2 s, respectively, P = 0.0064, two-way RM ANOVA, Bonferroni *post-hoc*; Fig. 4d). Interaction between tDCS and quadrant was statistically significant (F_3,42_ = 2.9, P = 0.045, two-way RM ANOVA).

TDCS-mice also showed significantly greater preference toward the novel object than controls in the NOR test (preference index: 73.0 ± 1.7% and 66.2 ± 1.1%, respectively; n = 8 for each group, P = 0.004, unpaired Student’s *t*-test; Fig. 4e).

### Anodal tDCS induces long-lasting changes in *Bdnf* expression by epigenetic mechanisms

Our next step was to determine the molecular mechanisms underlying the long-lasting effects of tDCS on hippocampal plasticity. Given the critical role of Bdnf in neuroplasticity-related events^22^,^23^, we checked if this neurotrophin was target of tDCS action.

**Figure 5:**
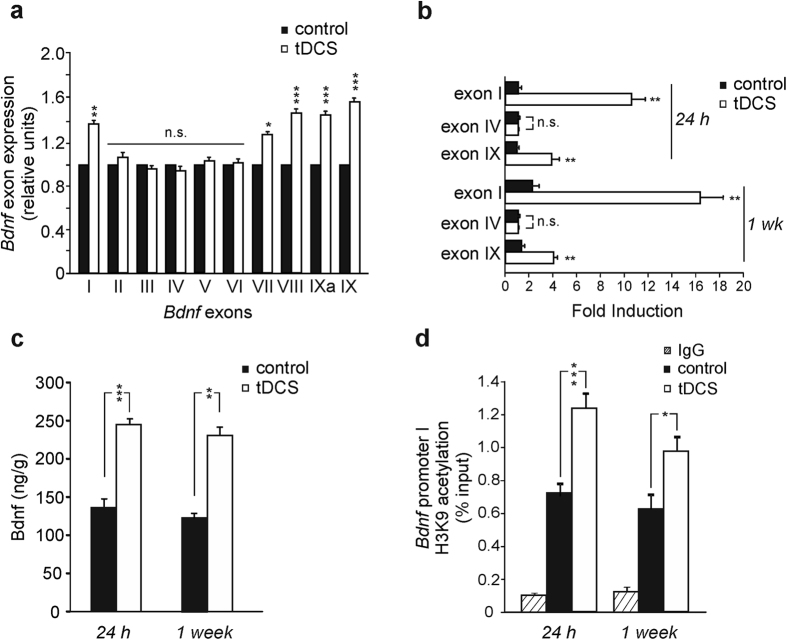
Anodal tDCS modulates *Bdnf* expression in the hippocampus through epigenetic mechanisms. (**a**) Bar graph showing results from semiquantitative RT-PCR analyses on exon specific mRNA levels of *Bdnf* 24 h after sham or anodal stimulation (n = 4 for each group; exon I: P = 0.0003; exon VII: P = 0.005; exon VIII: P = 8E-07; exon IXa: P = 5E-06; exon IX: P = 1E-07; exons II, III, IV, V and VI: P > 0.3 *vs.* controls, unpaired Student’s *t*-test or Mann-Whitney test). Analyses were performed in triplicate. (**b**) Changes in the expression of *Bdnf* exons I, IV, IX assessed by Real-Time qPCR. Gene expression was normalized to Gadph. Data represent mean values obtained from 3 mice for each group; experiment were performed in triplicate. Exon I: P < 0.001 at both 24 h and 1 week, Mann-Whitney test. Exon IV: P = 0.37 at 24 h and P = 0.7 at 1 week, unpaired Student’s *t*-test. Exon IX: P < 0.001 at both 24 h and 1 week, Mann-Whitney test. (**c**) Results from ELISA showing that Bdnf levels were increased 24 h and 1 week after tDCS (n = 4 mice/group/time point; P = 1.08E-06 at 24 h, unpaired Student’s *t*-test; P < 0.001 at 1 week, Mann-Whitney test). The assay was performed in duplicate. (**d**) Results from ChIP assays performed on hippocampal extracts of control and tDCS-mice showing tDCS-induced enhancement of histone H3 acetylation (H3K9ac) levels on promoter I of *Bdnf* both 24 h and 1 week after tDCS (P < 0.001 *vs.* controls, Mann-Whitney test). Data represent mean values obtained from 3 mice per group. PCR samples were run and analyzed in triplicate; data were obtained from two independent experiments. Data are expressed as percentage of input. Data are shown as mean ± s.e.m. *P < 0.05; **P < 0.001; ***P < 0.0001; n.s., not significant *vs.* control.

Semiquantitative RT-PCR analysis on hippocampal extracts obtained from mice sacrificed 24 h after stimulation revealed that anodal tDCS induced differential regulation of exon-specific *Bdnf* mRNAs, with increased levels of exons I, VII, VIII, IXa and IX transcripts in tDCS-mice compared to controls (n = 4 mice for each group; exon I: P = 0.0003; exon VII: P = 0.005; exon VIII: P = 8E-07; exon IXa: P = 5E-06; exon IX: P = 1E-07; exons II, III, IV, V and VI: P > 0.3 *vs.* controls, unpaired Student’s *t*-test or Mann-Whitney test; Fig. 5a and Supplementary Fig. 3a).

These results were validated by quantitative Real-Time PCR (qRT-PCR) analyses focused on *Bdnf* common 3′ coding exon IX and on exons I and IV that are highly responsive to neuronal activity^23^,^24^. Results confirmed tDCS-induced enhancement of *Bdnf* exon I and IX mRNAs (Fig. 5b). In particular, exon I mRNA levels were ~9.5-fold higher in tDCS-mice than in controls 24 h after stimulation (n = 3 mice for each group; P < 0.001, Mann-Whitney test) and ~7-fold higher 1 week after tDCS (n = 3 mice for each group P < 0.001, Mann-Whitney test; Fig. 5b). At both time points we also found enhanced expression of *Bdnf* exon IX in tDCS mice (~3.7-fold and ~3.0-fold higher than controls at 24 h and 1 week, respectively; P < 0.001, Mann-Whitney test; Fig. 5b). Conversely, the levels of *Bdnf* exon IV transcripts were similar in control and tDCS-mice confirming that the induction of this exon was not affected by tDCS (P = 0.37 at 24 h and P = 0.7 at 1 week, unpaired Student’s *t*-test; Fig. 5b).

Next, we checked whether increased expression of *Bdnf* transcripts following tDCS was accompanied by enhanced levels of the protein. Enzyme-Linked Immunosorbent Assay (ELISA) showed that 24 h as well as 1 week after tDCS hippocampal Bdnf levels were ~1.8 fold higher in tDCS-mice than in controls (n = 4 for each group/time point; P = 1.08E-06 at 24 h, unpaired Student’s *t*-test; P < 0.001 at 1 week, Mann-Whitney test; Fig. 5c). No changes in Bdnf levels were detected in non-stimulated areas such as the cerebellum ipsilateral to the stimulation side (Supplementary Fig. 4a). Further control experiments showed that other neuronal genes (e.g., NeuN) were not affected by tDCS. Indeed Western blot analysis of hippocampal extracts from tDCS and control mice revealed no significant differences in NeuN expression, thus suggesting that *Bdnf* is specific target of tDCS (Supplementary Fig. 4b,c).

With regard to the mechanism leading to enhanced *Bdnf* expression it is well known that neuronal plasticity and memory formation are tightly modulated by the epigenetic regulation of gene expression via histone modifications at particular gene promoter regions^33^. We hypothesized that anodal tDCS induced membrane depolarization mimicking neuronal activation and triggered epigenetic changes at *Bdnf*, thus favoring its transcription. Notably, enhancement of *Bdnf* exon I, IV and IX transcripts and increased acetylation of histones at promoter I have been previously reported following neuronal activation^23^,^24^,^34^,^35^,^36^. As such, we examined whether changes in H3 acetylation at lysine 9 (H3K9ac) on *Bdnf* promoter I occurred after tDCS.

ChIP assay performed on hippocampi from mice sacrificed 24 h after tDCS or sham stimulation (n = 3 mice for each group) revealed that tDCS significantly enhanced H3K9ac at *Bdnf* promoter I (+91%; P < 0.001 *vs.* controls, Mann-Whitney test; Fig. 5d; see also Supplementary Fig. 3b). ChIP experiments repeated 1 week after tDCS revealed increased H3K9ac at *Bdnf* promoter I in tDCS-mice at this time point too (+58%; P < 0.001 *vs.* controls; n = 3 for each group, Mann-Whitney test; Fig. 5d).

All together these results indicate that *Bdnf* expression in the hippocampus is induced by anodal tDCS and that enhanced acetylation at *Bdnf* promoter I is likely responsible for such effect.

**Figure 6:**
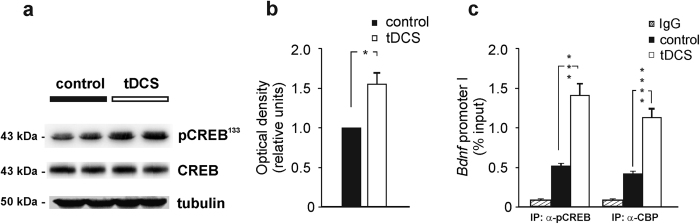
Activation of CREB/CBP pathway in the mouse hippocampus following anodal tDCS. (**a**) Representative Western blot analysis revealing increased phosphorylation of CREB at Ser133 by tDCS stimulation (see full scans in Supplementary Fig. 4). Expression levels of total CREB were unchanged. Experiments were performed in duplicate; samples were harvested from two independent experiments. (**b**) Band densitometry normalized to tubulin (P = 0.015, hippocampi from tDCS-mice *vs.* control [n = 5 tDCS-mice; n = 3 controls], unpaired Student’s *t*-test). (**c**) Results from ChIP assays performed on hippocampal extracts (n = 3 control mice and n = 3 tDCS-mice) showed that tDCS increased CREB binding (P < 0.0001, Mann-Whitney test) and CBP recruitment to *Bdnf* promoter I (P < 0.00001, Mann-Whitney test). *Bdnf* promoter I fragments were amplified from anti-CBP and anti-pCREB immunoprecipitated DNA or from the total chromatin input (TI) as quantitative control. PCR samples were run and analyzed in triplicate; data were obtained from two independent experiments. Data are expressed as percentage of input. Data are expressed as mean ± s.e.m. *P < 0.05; ***P < 0.0001; ****P < 0.00001.

We then asked whether tDCS effects might involve CREB/CREB-binding protein (CBP) signaling. Indeed, one pathway mediating neuronal activity-dependent histone acetylation involves activation of CREB that, once phosphorylated at Ser133 (pCREB^133^), binds the transcriptional coactivator CBP which acts as a histone acetyltransferase (HAT) thus altering chromatin structure of several genes including *CREB* itself and *Bdnf *36,37. We first checked whether anodal tDCS increased pCREB^133^ levels given that this stimulation has been reported to enhance intracellular Ca^2+^ signals^38^, that play a critical role in promoting CREB phosphorylation^39^. Hippocampi from control (n = 3) and tDCS-mice (n = 5) were collected two hours after tDCS and pCREB^133^ was assessed by Western blot analysis. Results showed that pCREB^133^ levels were significantly higher in hippocampal extracts from tDCS-mice than control mice (P = 0.015, unpaired Student’s *t*-test; Fig. 6a,b and Supplementary Fig. 5).

*Bdnf* promoter I contains a CRE region proximal to the transcription start site and within the sequence amplified by the primers we designed for ChIP experiments^23^ (Supplementary Table 2). ChIP analyses were then carried out to assess CREB binding and CBP recruitment to *Bdnf* promoters. Results showed tDCS-dependent increases in CREB binding to the promoter I (+300%; P < 0.0001, Mann-Whitney test) and CBP recruitment to promoter I (+181%; P < 0.00001, Mann-Whitney test; n = 3 mice for each group; same samples used to evaluate H3K9ac; Fig. 6c).

Collectively, these data suggest that anodal tDCS induced epigenetic changes at *Bdnf* promoters likely relying on a mechanism involving CREB activation, CBP recruitment and H3K9 acetylation.

**Figure 7:**
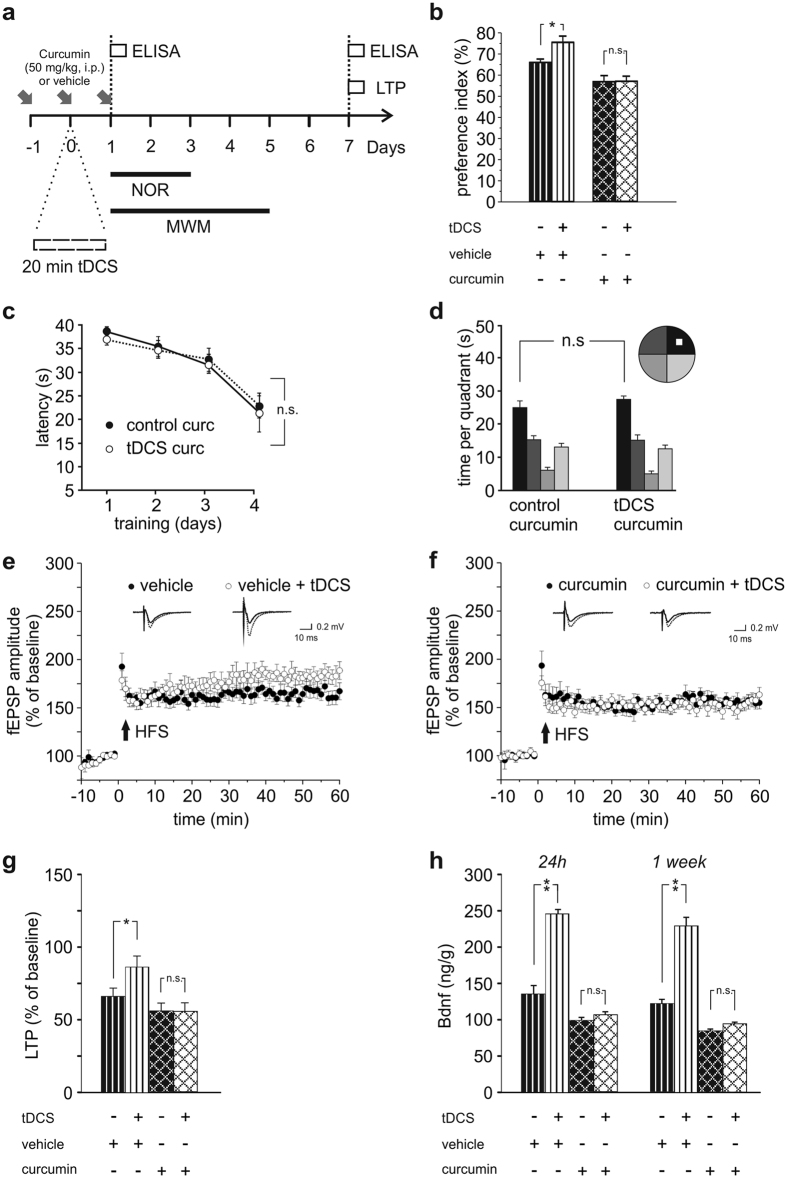
Inhibition of HAT activity hinders tDCS effects. (**a**) Schematic representation of the time schedule of the protocol in experiments with the HAT inhibitor, curcumin. (**b**) Preference toward the novel object in NOR paradigm (n = 7 for both vehicle-treated control and tDCS-mice; n = 8 for curcumin controls and n = 9 for curcumin tDCS-mice). Treatment with curcumin occluded anodal tDCS enhancement of memory in the NOR test (P = 0.95 curcumin injected tDCS mice *vs.* their relative control, two-way ANOVA, Bonferroni *post-hoc*; P = 0.005, vehicle-injected tDCS-mice *vs.* their controls, Bonferroni *post-hoc*). (**c**) In the MWM test, the latencies to navigate to the platform (F_1,45_ = 0.0007, P = 0.98 for overall acquisition; two-way RM ANOVA) and (**d**) the time spent in the target quadrant (P = 0.51; two-way RM ANOVA, Bonferroni *post-hoc*) were not significantly different in sham (n = 9) and tDCS-mice (n = 8) injected with curcumin. (**e,f**) Time course of LTP induced at CA3-CA1 synapses by HFS delivered at time 0 in hippocampal slices from sham and tDCS-mice injected with vehicle (**e**) or curcumin (**f**). Insets show representative fEPSPs at baseline (solid line) and during the last 5 min of LTP recording (dotted line). (**g**) Mean amplitude of fEPSPs recorded 60 min after HFS under the experimental conditions shown in e-f. In vehicle injected-mice tDCS enhanced LTP (P = 0.02, two-way ANOVA, Bonferroni *post-hoc*; n = 9 slices from 4 tDCS-mice and n = 12 slices from 4 control mice) whereas in curcumin-injected mice tDCS did not cause significant changes in LTP (P = 0.96, two-way ANOVA, Bonferroni *post-hoc*; n = 9 slices from 3 tDCS-mice and n = 9 slices from 3 control mice). (**h**) Curcumin treatment prevented tDCS-induced increases in Bdnf protein levels quantified 24 h and 1 week after tDCS (P < 0.001 vehicle-injected tDCS mice [n = 4] *vs.* their respective control [n = 4]; P = 0.4 curcumin-injected tDCS mice [n = 4] *vs.* their respective control [n = 4]; two-way ANOVA, Bonferroni *post-hoc*). Data reported in the first two columns at both time points are the same shown in Fig. 5c. Data are expressed as mean ± s.e.m. *P < 0.05, **P < 0.001.

To establish a causal link between enhanced acetylation and tDCS effects on plasticity we treated mice with either vehicle or the p300/CBP HAT inhibitor, curcumin^40^,^41^ (50 mg/kg body weight, i.p.; see Fig. 7a and Methods for injection schedule), and subjected animals to anodal tDCS or sham stimulation. Twenty-four hours after stimulation animals were engaged in the NOR and MWM (different set of animals were used for each behavioral test). Control experiments showed that curcumin treatment efficiently inhibited histone H3 acetylation (Supplementary Fig. 6).

In the NOR vehicle- and curcumin-injected mice subjected to sham-stimulation (i.e., control groups) showed preference index > 50% (Fig. 7b) but, consistent with a role of acetylation in memory formation^42^, curcumin-injected controls showed lower preference index value (curcumin-injected mice [n = 8]: 56.9 ± 2.5%; vehicle-injected mice [n = 7]: 67.9 ± 2.5%; P = 0.005, two-way ANOVA, Bonferroni *post-hoc*; Fig. 7b). Most importantly, curcumin treatment prevented the enhancement of recognition memory by anodal tDCS. Indeed, vehicle-injected tDCS-mice showed increased preference index compared to their respective sham-stimulated controls (75.5 ± 2.6% [n = 7] *vs.* 67.9 ± 2.5% [n = 7], respectively; P = 0.005, Bonferroni *post-hoc*) whereas the performance of curcumin-treated mice exposed to tDCS did not significantly differ from that of sham-stimulated, curcumin-injected mice (57.1 ± 2.1% [n = 9] *vs.* 56.9 ± 2.5% [n = 8], respectively; P = 0.95, Bonferroni *post-hoc*; Fig. 7b). Two-way ANOVA also confirmed significant effects of either tDCS (F_1,27_ = 5.34; P = 0.029) or curcumin (F_1,27_ = 43.46; P < 0.001) and interaction between tDCS and curcumin (F_1,27_ = 5.00; P = 0.034).

A separate cohort of mice injected with curcumin was tested in the MWM. In line with data obtained with the NOR, curcumin treatment hindered tDCS effects. Indeed no significant differences were found between sham-stimulated and tDCS-mice (n = 9 and n = 8, respectively) injected with curcumin as to: i) the latencies to navigate to the platform (F_1,45_ = 0.0007, P = 0.98 for overall acquisition, two-way RM ANOVA; Fig. 7c); ii) the time spent in the target quadrant (27.5 ± 3.2 s in tDCS-mice and 25.4 ± 3.4 s in controls; P = 0.51, two-way RM ANOVA, Bonferroni *post-hoc*; Fig. 7d). Statistical analysis revealed that overall performance of the two groups of mice in both acquisition and probe test phase was not significantly impaired by curcumin treatment (acquisition: main effect of days: F_3,45_ = 28.83, P < 0.001; probe test: main effect of quadrant: F_3,45_ = 22.36, P < 0.001; time in the target quadrant *vs.* the other quadrants: P < 0.05 for both groups), thus supporting the view that blockade of HAT activity specifically interferes with tDCS effects on cognitive performances.

In a different set of experiments we tested whether curcumin also prevented the effects of tDCS we observed 1 week after stimulation. To this aim we studied LTP in slices obtained from vehicle- or curcumin-treated mice sacrificed 1 week after stimulation (Fig. 7a). As expected, LTP was increased by tDCS in vehicle-treated animals (86.7 ± 9.0% [n = 9 slices from 4 tDCS-mice] *vs.* 65.9 ± 6.9% [n = 12 slices from 4 control mice]; P = 0.02, two-way ANOVA, Bonferroni *post-hoc*; Fig. 7e,g), whereas no significant differences were found between tDCS and sham-stimulated animals pre-treated with curcumin (56.5 ± 6.7% [n = 9 slices from 3 tDCS-mice] *vs.* 56.6 ± 5.5% [n = 9 slices from 3 control mice]; P = 0.96, two-way ANOVA, Bonferroni *post-hoc*; Fig. 7f,g). Two-way ANOVA also showed that both sham-stimulated controls displayed similar LTP values (P = 0.28, Bonferroni *post-hoc*) confirming the specific inhibitory effect of curcumin on tDCS-induced boosting of synaptic plasticity.

Additionally, blockade of HAT activity significantly reduced the tDCS-induced increase in hippocampal Bdnf levels we observed both 24 h and 1 week after stimulation (Fig. 7h). In particular, at 24 h Bdnf levels were: 104.7 ± 4.39 ng/g in curcumin-injected tDCS mice *vs*. 95.9 ± 3.37 ng/g in curcumin-injected sham-stimulated controls (n = 4 mice for each group; P = 0.41, two-way ANOVA, Bonferroni *post-hoc*) and 245.47 ± 8.95 ng/g in vehicle-injected tDCS mice *vs*. 132.34 ± 11.81 ng/g in vehicle-injected controls (n = 4 mice for each group; P < 0.001, Bonferroni *post-hoc*). Overall, two-way ANOVA showed a main effect of either tDCS (F_1,28_ = 67.87, P < 0.001) or curcumin (F_1,28_ = 143.43, P < 0.001) and an interaction between tDCS and curcumin (F_1,28_ = 49.78, P < 0.001).

Similar results were obtained 1 week after tDCS. At this time point Bdnf values were: 93.6 ± 2.60 ng/g in curcumin-injected tDCS-mice *vs.* 86.8 ± 2.37 ng/g in curcumin injected sham-stimulated controls (n = 4 mice for each group; P = 0.49; two-way ANOVA, Bonferroni *post-hoc*) and 228.0 ± 12.66 ng/g in vehicle-injected tDCS mice *vs*. 120.87 ± 6.22 ng/g in vehicle-injected controls (n = 4 mice for each group; P < 0.001; Bonferroni *post-hoc*; main effect of tDCS: F_1,28_ = 70.0, P < 0.001; main effect of curcumin: F_1,28_ = 153.16, P < 0.001; interaction: F_1,28_ = 54.45, P < 0.001).

**Figure 8:**
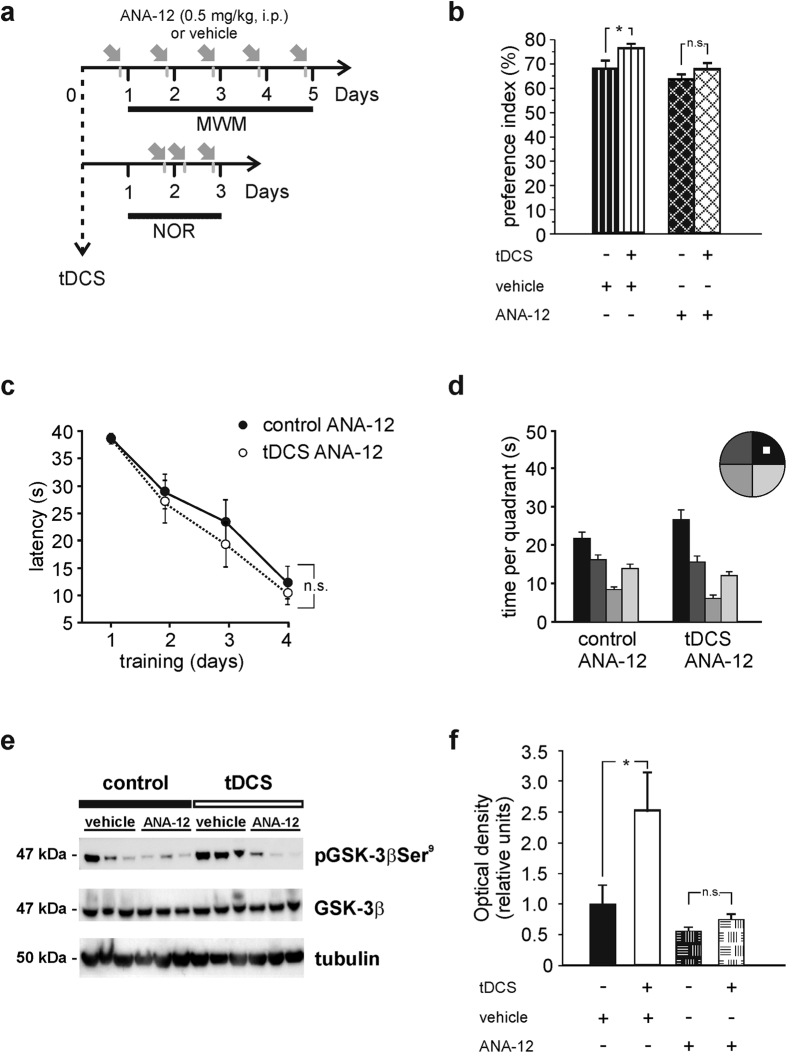
Blockade of the TrkB Bdnf receptor reduced tDCS facilitatory effects on memory. (**a**) Schematic representation of the time schedule of the protocol used in experiments involving the TrkB inhibitor, ANA-12. (**b**) Preference toward the novel object in the NOR paradigm was not increased in tDCS mice injected with ANA-12 (P = 0.008 vehicle-injected tDCS mice [n = 8] *vs.* their respective control [n = 8]; P = 0.18 ANA-12-injected tDCS mice [n = 8] *vs.* their respective control [n = 8]; two-way ANOVA, Bonferroni *post-hoc*). (**c,d**) ANA-12 injected tDCS-mice (n = 7) showed similar performances in the acquisition and probe test sessions of the MWM compared to sham-stimulated controls (n = 7). (**c**) Escape latency: F_3,36_ = 38.45, P < 0.001 for overall acquisition; P > 0.3 at each training days, tDCS-mice *vs.* control, two-way RM ANOVA, Bonferroni *post-hoc*. (**d**) Probe test: P = 0.19 *vs.* control; two-way RM ANOVA, Bonferroni *post-hoc*. (**e**) Representative Western blot analysis of hippocampal homogenates obtained from control (n = 3) and tDCS-mice (n = 3) revealing increased pGSK-3βSer^9^ by tDCS (P = 0.004; unpaired Student’s *t*-test). No changes in pGSK-3βSer^9^ were observed in hippocampi from ANA-12 injected tDCS-mice *vs.* ANA-12-injected control mice (P = 0.52; unpaired Student’s *t*-test; n = 3 for each group). (**f**) Band densitometry of pGSK-3βSer^9^/GSK-3β ratio normalized to tubulin. Experiments were performed in duplicate. Optical density values are expressed as fold-changes *vs.* control. Data are expressed as mean ± s.e.m. *P < 0.05; n.s., not significant *vs.* control.

To further understand the molecular mechanisms underlying tDCS effects on plasticity we sought to establish a causal link between the increased levels of Bdnf and tDCS effects. Mouse treatment with the inhibitor of the Bdnf receptor TrkB, ANA-12^43^ (0.5 mg/kg body weight, Fig. 8a), reduced the facilitatory effects of tDCS on learning and memory assessed by NOR and MWM tests. In the NOR test performed on vehicle-injected animals the preference index was 68.0 ± 3.2% and 76.6 ± 1.3% in sham-stimulated and tDCS-mice, respectively (n = 8 for each group, P = 0.008 two-way ANOVA, Bonferroni *post-hoc*); in ANA-12-treated animals the preference index was 63.6 ± 2.0% and 67.8 ± 2.3% in sham-stimulated and tDCS-mice, respectively (n = 8 for each group; P = 0.18, two-way ANOVA, Bonferroni *post-hoc*; Fig. 8b). Two-way ANOVA showed that recognition memory was not significantly impaired by ANA-12 treatment (P = 0.16 vehicle-injected *vs.* ANA-12-injected control groups, Bonferroni *post-hoc*).

In the MWM treatment with ANA-12 did not affect overall performance in all mice (n = 7 tDCS-mice and n = 7 sham-stimulated mice; F_3,36_ = 38.45, P < 0.001 for overall acquisition; two-way RM ANOVA) but prevented the tDCS-induced enhancement in learning the location of the hidden platform (P > 0.3 at each training days; tDCS-mice *vs.* controls, two-way RM ANOVA, Bonferroni *post-hoc*; Fig. 8c). Consistently, memory performance of stimulated mice treated with ANA-12 was not significantly improved by tDCS (24.7 ± 1.9 s *vs.* 22.2 ± 2.0 s in control mice; P = 0.19, two-way RM ANOVA, Bonferroni *post-hoc*; Fig. 8d). Statistical analysis showed that spatial memory was not impaired by ANA-12 treatment (probe test: main effect of quadrant: F_3,48_ = 51.94, P < 0.001; time in the target quadrant *vs.* the other quadrants: P < 0.005 for both groups; Fig. 8d).

These results strongly support our hypothesis that increased histone acetylation promoting *Bdnf* transcription plays a major role in anodal tDCS-induced enhancement of synaptic plasticity.

To investigate molecular pathways downstream of Bdnf/TrkB signaling potentially responsible for tDCS-induced increased synaptic plasticity we examined phosphorylation of Glycogen synthase kinase-3β (GSK-3β), that is a key regulated substrate of TrkB. Indeed, GSK-3β is highly expressed in the rodent hippocampus where it has been involved in the modulation of synaptic plasticity^44^. Notably, TrkB activation produces GSK-3β inhibition by its phosphorylation at Serine 9 leading to enhanced LTP^45^. Hippocampi from control (n = 7) and tDCS-mice (n = 7) were collected 24 hours after tDCS and pGSK-3βSer^9^ levels were assessed by Western blot. Results showed that pGSK-3βSer^9^ levels were significantly higher in hippocampal extracts from tDCS-mice than control mice (P = 0.004, unpaired Student’s *t*-test; Fig. 8e,f). Interestingly, the tDCS-induced enhancement of pGSK-3βSer^9^ was not observed in tissues obtained from mice treated with ANA-12 (n = 3 for each group; P = 0.52, Fig. 8e,f). The ability of ANA-12 to prevent TrkB activation in our experimental model was confirmed by Western blot analysis showing that the levels of TrkB phosphorylated at Tyrosine816 was reduced in hippocampi from mice treated with ANA-12 compared to vehicle-injected mice (n = 5 ANA-12 injected mice *vs.* n = 4 vehicle-injected mice; P = 0.0006; unpaired Student’s *t*-test; Supplementary Fig. 7).

## Discussion

The present study elucidated the impact of anodal tDCS on hippocampal synaptic plasticity and shed light on molecular mechanisms underlying the learning and memory improvement we observed in the stimulated mice.

Results showed that LTP was increased in hippocampal slices from mice subjected to anodal tDCS. tDCS-mice also performed better in the MWM and NOR tests. These effects were seen within hours after tDCS and, remarkably, they persisted 1 week. TDCS effects were due to enhanced exon-specific mRNA expression of *Bdnf.* The hippocampi of tDCS-mice showed increased pCREB^Ser133^ levels along with epigenetic modifications including CREB binding to the *Bdnf* promoter I, recruitment of the HAT CBP leading to enhanced H3K9ac on *Bdnf* promoter I. These results provide novel insights into molecular mechanisms underlying tDCS effects whose understanding is required for a rational use of this technique to potentiate/ameliorate brain functions.

The tDCS intensity we used was below the safety limits of this stimulation^27^,^38^, as such, no morphological alterations were found in brain tissues subjected to tDCS. The montage we used has been widely employed in rodents and it has been proven to specifically target the cortical brain area under the stimulating electrode and to reproduce results of tDCS in humans^46^,^47^,^48^. Furthermore, previous studies using similar devices in rodents demonstrated that the path of current flowing between the electrodes penetrates not only the cortex but also sub-cortical structures including the hippocampus^20^,^49^,^50^. Although tDCS likely affected the brain cortex underneath the stimulation electrode, the neurophysiological, behavioral and molecular changes investigated in our study were all related to hippocampal function. Indeed, anodal tDCS enhanced LTP at hippocampal CA3-CA1 synapses and improved spatial and recognition memory assessed by two validated behavioral tests of hippocampal-dependent memory, i.e., MWM and NOR^51^,^52^.

Our finding that LTP values in the hippocampus contralateral to the stimulating electrode were not significantly different from those of the ipsilateral one might be related to input conveyed by hippocampal commissural connections.

On the other hand in control experiments no changes in hippocampal LTP were found when stimulation was delivered over the primary motor cortex, thus supporting the specificity of the observed effects. The finding that cathodal tDCS induced opposite effects on hippocampal LTP confirms our previous observations suggesting polarity-dependent DCS effects on brain plasticity^19^, and further supports the specificity of the stimulation protocol we used. We attributed the effects of tDCS to modifications of synaptic plasticity rather than to changes in synaptic transmission since we found no significant differences between data collected in slices from tDCS- and sham-stimulated mice as to: i) input-output curves obtained by plotting fEPSP amplitude against stimulus intensities or against presynaptic fiber volleys, indicating no changes in basal synaptic transmission; ii) PPR, indicating that glutamate release probability at CA3-CA1 synapses was unaffected by tDCS; iii) AMPA/NMDA ratio, indicating that tDCS *per se* did not elicit changes in synaptic strength as also reported by Fritsch *et al.*^18^ in the motor cortex. Our findings are in agreement with data recently reported by Rohan *et al.*^20^ showing that anodal tDCS enhanced hippocampal LTP in rats without affecting basal synaptic transmission. The same authors also reported a transient effect of tDCS on PPR that did not parallel LTP changes: indeed increased PPR was only found in rats subjected to 30 min recovery after tDCS but not in those housed for 24 hours post-stimulation. In our experimental model no changes in PPR were found, probably because our recordings fell out of the time window of the transient changes observed by Rohan and coworkers and/or because of differences related to the different species we used.

Our findings are supported by previous reports suggesting that: i) DC stimulation modulates LTP elicited by other NIBS techniques^53^,^54^,^55^; ii) tDCS influences LTP-related learning and memory processes^5^; iii) tDCS effects on motor and cognitive functions are greater when stimulation is paired with “activity” engaging the subject’s stimulated brain area^56^.

Remarkably, we found that one 20-min tDCS elicited electrophysiological and behavioural effects clearly detectable 1 week later. These findings suggested us the engagement of molecular pathways retaining memory of stimulation and the possible involvement of epigenetic regulation of plasticity-related genes. *Bdnf* was the most likely candidate since it is highly activity-regulated through both transcriptional and epigenetic mechanisms especially in the hippocampus, and the neurotrophin Bdnf is a key regulator of synaptic plasticity^34^. Of note, several lines of evidence indicate that the regulation of *Bdnf* activity is a correlate of hippocampus-dependent learning *in vivo*^21^ and converging studies implicate Bdnf in the responses to tDCS in humans and rodents^18^,^57^,^58^.

ChIP experiments performed on the hippocampi of control and tDCS-mice revealed that anodal stimulation increased H3K9ac at *Bdnf* promoter I. This effect was observed 24 h as well as 1 week after tDCS and was paralleled by enhanced expression of *Bdnf* exon I and common 3′ coding exon IX specific mRNAs. Consistently with our results histone acetylation levels on *Bdnf* promoters, especially promoter I, have been shown to affect LTP and long-term memories^59^. Consequently, disruption of HAT impairs several forms of learning and memory, including novel object recognition, spatial learning in the MWM and fear conditioning^60^. Of note, recent work revealed a correlation among impaired LTP, reduced histone acetylation and altered Bdnf/TrkB signaling during aging. Such dysfunctional plasticity and impaired epigenetic pathways have also been observed in the prefrontal cortex and hippocampus of mouse models of depression and stress^25^,^61^. These data highlight the potential relevance of our findings for using tDCS as tool for epigenetic intervention strategy to treat diseases associated to impaired synaptic plasticity.

The link between the electrophysiological/functional outcomes of anodal tDCS and epigenetic changes at *Bdnf* promoters is supported by the inhibitory effects we observed following treatment with curcumin that is a specific p300/CBP HAT inhibitor at the concentration we used^41^. In curcumin-treated mice tDCS failed to induce significant increases in: i) learning and memory at both MWM and NOR; ii) LTP at CA3-CA1 synapses; iii) hippocampal Bdnf levels.

The role of Bdnf pathway activation in the improved learning and memory we observed in tDCS-mice is demonstrated by results showing that *in vivo* administration of the Bdnf TrkB receptor inhibitor, ANA-12, abolished the facilitatory effects of tDCS in both MWM and NOR.

Of note we found that tDCS enhanced GSK-3β phosphorylation at Ser 9 inhibiting the enzyme activity^44^,^45^ and this effect was prevented by ANA-12, indicating its dependence on activation of TrkB. Therefore inhibition of GSK-3β activity is likely one of the mechanism contributing to the LTP enhancement we found following tDCS. Interestingly, a recent study demonstrated that exogenous application of the Val66pro-form but not the Met66pro-form of Bdnf to hippocampal slices inhibited LTP and facilitated long-term depression through dephosphorylation of GSK-3β at Ser 9^62^. This study takes on particular relevance in light of our findings given that Bdnf Val66Met polymorphism is known to affect responses to tDCS in humans^57^.

Collectively, these data support the view that anodal tDCS boosts synaptic plasticity by enhancing *Bdnf* expression in response to LTP induction protocols and learning. Nevertheless, other plasticity-related genes, that are worth studying in future research, might cooperate with Bdnf in mediating tDCS action. If operating in other brain areas, the molecular mechanism we uncovered here is a plausible substrate for tDCS action on cognitive and motor functions documented in the literature. It also provides the rationale for using tDCS in combination with other training/stimulation protocols converging onto the same target genes. Within this clinical perspective elucidation of the molecular machinery whereby tDCS induces epigenetic changes at *Bdnf* favoring its transcription is a very critical issue. In this regard we demonstrated that anodal tDCS increases CREB phosphorylation at Ser133 and CREB binding to *Bdnf* promoter I, which is hyperacetylated following tDCS. Tabuchi *et al.*^63^ reported that a CRE-like response element contributes to the activity-mediated induction of rat *Bdnf* promoter I whereas Pruunsild and coworkers^24^ showed that mutation in CRE did not alter the induction of human *Bdnf*, thus suggesting differential regulation of promoter I activity by CREB at transcriptional level in humans and rodents. Our study linked tDCS-induced CREB binding at CRE element on promoter I to epigenetic changes likely involving recruitment of the HAT CBP at the same region. TDCS-dependent upregulation of *Bdnf* exon I mRNA is fully consistent with enhanced transcriptional activity at promoter I. Interestingly, exon IV transcript, that is the most induced *Bdnf* mRNA in response to neuronal activity, was not sensitive to tDCS, supporting the view that this non-pharmacological brain-treatment elicits exon-specific modulation of *Bdnf*.

Our data involving CREB/CBP in tDCS-mediated effects on plasticity are consistent with the critical role of CBP in CREB activity and epigenetic regulation of learning and memory^37^. Furthermore, they provide a plausible, yet not necessarily unique, mechanism for the enhanced acetylation and increased exon-specific *Bdnf* mRNA expression we observed following tDCS.

**Figure 9:**
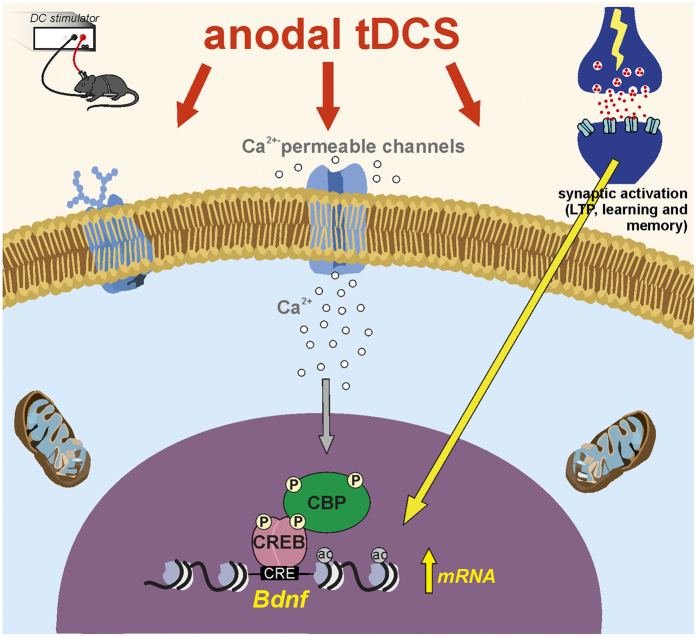
Model of anodal tDCS-induced chromatin remodeling leading to enhanced hippocampal synaptic plasticity. Based on our data we propose that transient increase in intracellular Ca^2+^ during tDCS initiates molecular cascades leading to persistent changes in chromatin structure of *Bdnf*. These include the phosphorylation of CREB, its binding to *Bdnf* promoter I and recruitment of CBP. CBP, in turn, promotes H3K9 acetylation of *Bdnf* (specifically at promoter I) because of its HAT activity. As a result, stimuli such as LTP induction protocol in slices or learning and memory *in vivo* are more effective in promoting transcription of *Bdnf* previously primed by anodal tDCS.

As for the molecular events linking tDCS to the above reported cascades leading to enhanced acetylation, it is reasonable to hypothesize the involvement of increased Ca^2+^ levels. TDCS effects have been directly or indirectly associated with membrane depolarization-dependent increases in intracellular Ca^2+^ levels via NMDAR and voltage-gated calcium channel activation^20^,^38^,^64^. Increased Ca^2+^ levels could initiate molecular pathways leading to HAT activation via CREB/CBP (Fig. 9).

Enhanced acetylation at the promoters of *Bdnf* and other potential target genes provides a molecular substrate whereby tDCS influences synaptic plasticity in the hippocampus leading to enhanced LTP and cognitive performances. This mechanism might likely be engaged in other cortical areas targeted by tDCS protocols used in humans. Given that Bdnf and epigenetic regulation of gene expression are considered promising targets to potentiate synaptic plasticity in physiology and pathology our data ground and support the use of tDCS as viable therapeutic approaches in neuropsychiatric and neurodegenerative diseases.

## Methods

### Ethics and Animal Use Statement

Male C57bl/6 mice (30–45 day-old) were used and randomly assigned to two main groups: (i) tDCS-treated and (ii) sham-stimulated animals (controls). Different groups of mice were used for each experimental test and time point from tDCS.

All animal procedures were approved by the Ethics Committee of the Catholic University and were fully compliant with Italian (Ministry of Health guidelines, Legislative Decree No. 116/1992) and European Union (Directive No. 86/609/EEC) legislations on animal research.

The methods were carried out in strict accordance with the approved guidelines.

The animals were housed under a 12-h light-dark cycle at room temperature (RT: 19–22 °C). Efforts were made to limit the number of animals used and to minimize their suffering.

### Electrode implantation and transcranial direct current stimulation

For tDCS stimulation we adopted an electrode montage widely used and well validated in both rat and mouse models consisting in an unilateral epicranial electrode arrangement^48^,^49^. Specifically, the active electrode consisted of an epicranial implanted tubular plastic jack (inner area = 6.25 mm^2^; model GB3305-00, Star electronic SPA, Italy) filled with saline solution (0.9% NaCl) just prior to stimulation; the counter electrode was a conventional rubber-plate electrode surrounded by a wet sponge (5.2 cm^2^; Physiomed Elektromedizin AG, Germany) applied over the ventral thorax by a custom corset. According to the literature this unipolar arrangement prevents currents from bypassing between two juxtaposed epicranial electrodes.

For the epicranial electrode implant, animals were anesthetized with a cocktail of ketamine (70 mg/Kg, intramuscular injection [i.m.]) and medetomidine (1 mg/Kg, i.m.) and temperature during surgery was maintained at 37 °C. The scalp and underlying tissues were removed and the electrode was implanted using a carboxylate cement (3 M ESPE, Durelon, 3 M Deutschland GmbH, Germany). The centre of the active electrode was positioned on the skull over the left hippocampal formation 1 mm left and 1 mm posterior to bregma^26^. After surgery, all animals were allowed to recover for 3–5 days before undergoing tDCS.

TDCS was applied to awake and freely moving mice at a current intensity of 350 μA for 20 min using a battery-driven, constant current stimulator (Stimulus Isolator, model: A385, World Precision Instruments, USA). This intensity corresponded to a current density of 56 μA/mm^2^ (0.35 mA/0.0625 cm^2^) that was within the range of that used previously in rats^48^ (57.1 μA/mm^2^) or mice^38^,^49^ (55.5 μA/mm^2^). The current intensity was ramped for 10 s instead of switching it on and off directly to avoid a stimulation break effect. The animals were observed during tDCS and *in vivo* experiments to determine possible abnormal behaviors related to the stimulation. Control animals received sham stimulation in which no current was applied but the animal underwent the same manipulations as in the stimulation condition. We chose to apply tDCS over the left hippocampus given that, experimental evidences suggest that long-term memory processing is strictly dependent on the left hemisphere^65^.

Experimental design and protocol timeline of experiments are summarized in Figs 1, 7a and 8a. Electrophysiological, molecular and behavioral analyses were performed either at short-term (2–24 h) and long-term (7 days) intervals from sham stimulation or tDCS, to assess the duration of effects elicited by a single tDCS session.

Investigators were blinded to the identity of the groups during experiments and analysis.

### Histological processing

Histological evaluation was carried out to detect possible current-induced neurotrauma (e.g., oedema, necrosis, haematoma, cellular alterations). At the end of the stimulation session animals were deeply anesthetized with a cocktail of ketamine (80 mg/Kg, i.m.) and medetomidine (1 mg/Kg, i.m.) and perfused transcardially with saline followed by a fixative containing 4% paraformaldehyde in 0.1 M PBS. After post-fixation, brains were removed from the skulls and stored at 4 °C in a high sucrose solution (30% sucrose in 0.1 M PBS) for 2 days. A vibratome (VT1000S, Leica Microsystems) was used to collect serial coronal 40-μm thick sections containing the hippocampus. All sections were further processed for hematoxylin-eosin staining. Images were acquired with Olympus BX3-CBH microscope.

### Electrophysiology

Whole-cell patch-clamp and field recordings were performed on hippocampal coronal slices (300- and 400-μm-thick, respectively) as previously described^28^,^66^,^67^. Briefly, mice were anesthetized by halothane inhalation (Sigma) and decapitated. The brain was rapidly removed and put in ice-cold cutting solution (in mM: 124 NaCl, 3.2 KCl, 1 NaH_2_PO_4_, 26 NaHCO_3_, 2 MgCl_2_, 1 CaCl_2_, 10 glucose, 2 sodium pyruvate, and 0.6 ascorbic acid, bubbled with 95% O_2_-5% CO_2_; pH 7.4). Slices were cut with a vibratome (VT1000S) and incubated in artificial cerebrospinalfluid (aCSF; in mM: 124 NaCl; 3.2 KCl; 1 NaH_2_PO_4_, 1 MgCl_2_, 2 CaCl_2_; 26 NaHCO_3_; 10 glucose; pH 7.4; 95% O_2_-5% CO_2_) at 32 °C for 60 min and then at RT until use. Slices containing the left (i.e., stimulated) hippocampus were used for subsequent analyses.

Slices were transferred to a submerged recording chamber and continuously perfused with aCSF (flow rate: 1.5 ml/min). The bath temperature was maintained at 30–32 °C with an in-line solution heater and temperature controller (TC-344B, Warner Instruments). Identification of slice subfields and electrode positioning were done with 4× and 40× water immersion objectives on an upright microscope equipped with differential interference contrast and fluorescence optics under infrared illumination (BX5IWI, Olympus) and video observation (C3077-71 CCD camera, Hamamatsu Photonics).

All recordings were made using MultiClamp 700 A amplifier (Molecular Devices). Data acquisition and stimulation protocols were performed with the Digidata 1440 Series interface and pClamp 10 software (Molecular Devices). Data were filtered at 1 kHz, digitized at 10 kHz, and analyzed both online and offline.

Field recordings were made using glass pipettes filled with aCSF (tip resistance 2–5 MΩ) and placed in the stratum radiatum of the CA1 region. FEPSPs were evoked by stimulation of the Schaffer collaterals with a bipolar tungsten electrode (FHC, USA) connected to a S11 Grass stimulator (Grass Instruments).

The stimulation intensity that produced one-third of the maximal response was used for the test pulses, LTP induction and paired-pulse facilitation protocols. The fEPSP amplitude was measured from baseline to peak.

Before the LTP induction protocol, to check for a possible effect of tDCS on the basal synaptic transmission, I/O curves were obtained i) by recording fEPSPs induced by presynaptic stimulation at intensities ranging from 0 to 50 V (in 5 V-steps); ii) by plotting fEPSP amplitudes against presynaptic fiber volley amplitudes and then comparing the angular coefficients of I/O slopes. On the same synapses paired-pulse facilitation was also examined at 50 ms interpulse intervals.

For LTP recordings, stable baseline responses to test stimulations (once every 20 s for 10 min) were recorded and then HFS was delivered (4 trains of 50 stimuli at 100 Hz, 500 ms each, repeated every 20 s)^19^,^68^. Responses to test pulse were recorded every 20 s for 60 min to assess LTP. LTP magnitude was expressed as the percentage change in the mean fEPSP peak amplitude normalized to baseline values (i.e., mean values for the last 5 minutes of recording before HFS, taken as 100%). The occurrence of LTP was statistically verified (paired Student’s *t*-test) by comparing mean fEPSP amplitude measured at 55–60 min after HFS relative to baseline responses (Supplementary Table 1). Slices were prepared soon after the delivery of stimulation protocol and LTP recordings started ~2 hours after tDCS or sham stimulation.

Whole-cell voltage-clamp recordings were performed to measure the AMPA/NMDA ratio, expressed as the peak AMPA evoked post-synaptic currents (EPSCs, mean values of 30–50 events) at −70 mV divided by the peak NMDA EPSCs (mean values of 20–40 events) measured at 50 ms after the onset of the dual EPSC component at +40 mV.

For this set of recordings the electrodes were filled with internal solution containing (in mM): 135 CsMeSO_3_, 8 NaCl, 10 HEPES, 0.25 EGTA, 2 Mg_2_ATP, 0.3 Na_3_GTP, 0.1 spermine, 7 phosphocreatine and 5 QX-314 (pH: 7.25–7.30; 294–298 mOsm/l). We monitored the access resistance and membrane capacity before and at the end of the experiments to ensure recording stability and the health of studied cells. Recordings were considered stable when the series and input resistances, resting membrane potential, and stimulus artifact duration did not change > 20%.

### Behavioral tests

A set of animals was tested in the MWM and NOR that are the most widely used and standardized tests to investigate hippocampal-dependent learning and memory^51^,^52^. To better link changes of learning and memory performances to tDCS we avoided challenging the animals in more than one behavioral test. Therefore different groups of mice were used for each behavioral test and time point from tDCS (Fig. 1).

Mice were trained in the MWM to find a platform hidden 1 cm below the surface of a pool (127 cm in diameter) filled with water made opaque with white nontoxic paint. The acquisition training session started 4 days before the test session (probe test) and consisted of six trials a day for 4 consecutive days, during which the animals were allowed to reach the platform within 40 s. Starting points were changed daily and different starting points were used for each trial. A trial lasted either until the mouse had found the platform or for a maximum of 40 s. Mice rested on the platform for 10 s after each trial. Time (latency) needed to navigate to the platform and swim path length were recorded by an automated video tracking system (Panlab Harvard Apparatus). The probe test session was performed 24 h after the last day of the training. In this session, the platform was removed and each mouse was allowed to swim for 60 s; the time spent in each quadrant was measured^69^.

The NOR protocol lasted three consecutive days including a familiarization phase, a training phase and a test phase. On the 1^st^ day, mice were individually submitted to a single familiarization session of 10 min, during which they were introduced into the empty arena (45 × 45 cm). On the 2^nd^ day, animals were submitted to a single 10 min session (training phase) during which two identical objects were placed in a symmetric position from the centre of the arena. An explorative behavior was scored when the head of the animal was facing close (>2 cm away) to the object or any part of the body except the tail was touching the object. The time spent exploring each object was recorded. The animals were returned to their home cages immediately after training. On the 3^rd^ day, during the test phase, one of the familiar objects used during the training was replaced by a novel object and the animals were allowed to explore freely for 10 min. All objects were balanced in terms of physical complexity and were emotionally neutral. The open-field and the objects were cleaned by 70% alcohol after each session to avoid possible odorant cues. Preference index, i.e., the ratio of the amount of time spent exploring any one of the two items or the novel object over the total time spent exploring both objects, was used to measure recognition memory^70^.

### RNA extraction and cDNA synthesis

Total RNA was extracted from the left hippocampi of control and tDCS-mice using QIAzol Lysis reagent (Qiagen) according to the manufacturer’s instructions. RNA sample integrity and concentrations were evaluated with the BioPhotometer plus (Eppendorf, Germany). Reverse transcription reactions were performed on equal amounts of RNA (2 μg) with a high capacity cDNA reverse transcription kit (Applied Biosystems, USA).

### Semiquantitative RT-PCR and quantitative Real-Time PCR

Semiquantitative PCR of the cDNA was performed using Taq Polymerase (Fischer) and primers described by Aid *et al.*^23^ (Supplementary Table 3). The values of the control samples were set to 1.0, and the others were expressed as fold changes relative to the controls. Analyses were performed in triplicate on the left hippocampi obtained from controls and tDCS-mice.

Quantitative Real-Time PCR (qRT-PCR) amplifications were performed using Power SYBR® Master Mix on AB7500 instrument (Life Technologies) according to the manufacturer’s instructions. The thermal cycling profile featured a pre-incubation step of 94 °C for 10 min, followed by 40 cycles of denaturation (94 °C, 15 s), annealing (55–57 °C, 30 s), and elongation (72 °C, 20 s). Melting curves were subsequently generated by heating amplified products at 94 °C for 15 s, cooling to 50 °C for 30 s, followed by slow heating to 94 °C in increments of 0.5 °C. Melting-curve analyses confirmed that only single products had been amplified. The primer sequences coding the *Bdnf* exons I, IV and IX are shown in Supplementary Table 4. All data were normalized by reference to the amplification levels of the glyceraldehyde-3-phosphate dehydrogenase (Gapdh) gene; a reference dye was included in the SYBR master mix. The thresholds calculated by the software were used to calculate specific mRNA expression levels using the cycle-at-threshold (Ct) method, and all results are expressed as fold changes (compared to control) for each transcript, employing the 2^-ΔΔCt^ approach.

### Chromatin Immunoprecipitation

Chromatin immunoprecipitation (ChIP) assays were performed as previously described with minor modifications^69^. Animals were sacrificed 24 h or 1 week after the end of the stimulation protocol (Fig. 1). Mice were anesthetized with a cocktail of ketamine (80 mg/Kg, i.m.) and medetomidine (1 mg/Kg, i.m.) and transcardially perfused with an oxygenated Ringer’s solution (pH: 7.3), followed by 4% freshly depolymerized paraformaldehyde in 0.1 M PBS (pH:7.4). The brain was post-fixed overnight at 4 °C, and then transferred to a solution of 30% sucrose in PBS for 2 days. Coronal brain sections (45-μm-thick) containing hippocampi were then cut with a vibratome (VT1000S) and floated in ice-cold PBS.

Left hippocampi were isolated under optic microscope and minced through a 10 ml-syringe with decreasing needle size (18 to 22 gauge). Tissue lysate was resuspended in 200 μl lysis buffer containing SDS (1%), Tris-HCl (50 mM, pH 8.1), and EDTA (10 mM) and sonicated on ice with six 10-s pulses with a 20-s interpulse interval. Sample debris was removed by centrifugation, and supernatants were precleared by incubation with protein-G Sepharose 4B beads (Sigma) for 1 h at 4 °C. Beads were collected by centrifugation and supernatants were subjected to immunoprecipitation. Two μg of specific antibody (anti-pCREBSer^133^, anti-acetyl histone H3K9 from Millipore, anti-CBP from Abcam) or control IgG were added overnight at 4 °C. Immune complexes were collected by incubation with protein-G Sepharose 4B beads for 2 h at 4 °C and subjected to a series of seven sequential washes. Immune complexes were eluted from beads by vortexing in elution buffer (1% SDS and NaHCO_3_ 0.1 M; pH 8.0). NaCl was added (final concentration 0.33 M), and cross-linking was reversed by incubation overnight at 65 °C. DNA fragments were purified by using the PCR DNA fragments purification kit (Geneaid). The primer sequences for promoter I were designed on the basis of mouse *Bdnf* structure described by Aid *et al.*^23^ (Supplementary Table 2).

PCR conditions and cycle numbers were determined empirically and each PCR reaction was performed in triplicate. Data are expressed as percentage of input calculated by the “Adjusted input value” method according to the manufacturer’s instructions (ThermoFisher Scientific ChIP Analysis). In particular to calculate the Adjusted input the Ct value of input was subtracted by 6.644 (i.e., log2 of 100). Next, the percent input of control and tDCS samples was calculated using the formula: 100*2^(Adjusted input – Ct(ChIP). In the same way, the percent input of IgG samples was calculated using the formula 100*2^(Adjusted input – Ct(IgG).

Semiquantitative PCR amplification of *Bdnf* regulatory sequences performed on chromatin immunocomplexes showed that tDCS induced enhancement of H3K9 acetylation at promoter I but not at promoters II, III, and IV, thus supporting specificity of tDCS effects (Supplementary Fig. 3b and Supplementary Table 3; same samples used for ChIP assays showed in Fig. 5d).

### Western Immunoblot

Total proteins were extracted from the left hippocampi of control and tDCS-mice sacrificed 2 h after stimulation, by using ice cold RIPA buffer (Pierce; 50 mM Tris, 150 mM NaCl, 1 mM EDTA, 1% DOC, 1% Triton X-100, 0.1% SDS, and 1 × protease, phosphatase-1, and phosphatase-2 inhibitor cocktails [Sigma]). The lysate was centrifuged (20,000 × g, 30 min, 4 °C), and a 5-μl aliquot of the supernatant was assayed to determine the protein concentration (microBCA kit, Pierce). SDS-PAGE reducing sample buffer was added to the supernatant, and samples were heated to 95 °C for 5 min. Protein lysates (40–60 μg) were loaded onto 10% Tris-glycine polyacrylamide gels for electrophoretic separation. Colorburst^TM^ Electrophoresis markers (Sigma) were used as molecular mass standards. Proteins were then transferred onto nitrocellulose membranes at 100 V for 2 h at 4 °C in transfer buffer containing 25 mM Tris, 192 mM glycine, 0.1% SDS, and 20% methanol. Membranes were incubated for 1 h with blocking buffer (5% skim milk in TBST), and then incubated overnight at 4 °C with primary antibodies directed against one of the following proteins: pCREBSer^133^; CREB; pGSK-3βSer^9^; GSK-3β; panH3ac; NeuN; actin and tubulin (1:1,000). After three 10-min rinses in TBST, membranes were incubated for 1 h at RT with HRP-conjugated secondary antibodies (1:2,500). The membranes were then washed, and the bands were visualized with an enhanced chemiluminescence detection kit (GE Healthcare, UK).

Protein expression was evaluated and documented by using UVItec Cambridge Alliance. Experiments were performed in triplicate.

Images of Western blots have been cropped for presentation and full-size images are shown in Supplementary Figs 5 and 8. Antibodies are listed in Supplementary Table 5.

### Curcumin and ANA-12 administration

Curcumin (Sigma) was diluted in DMSO^40^ (i.e., vehicle). Mice received curcumin 50 mg/kg or vehicle via intraperitoneal (i.p.) injection. Mice were randomly assigned to sham or tDCS-group. Curcumin or vehicle was given 24 h prior to tDCS or sham stimulation, soon before stimulation and the following day (Fig. 7a). Two hours later some animals were engaged in: i) the habituation session of the NOR test; ii) the 1^st^ training session of the MWM; iii) tissue explant for ELISA assay. A week after stimulation (i.e., 6 days after the last curcumin injection) the other mice were used for: i) slice preparation for LTP recordings; ii) tissue explant for ELISA assay (Fig. 7a).

For experiment with ANA-12, mice were divided in two groups receiving sham stimulation or anodal tDCS and subjected to NOR or MWM tests. A volume of 10 μl/g body weight was i.p. injected for vehicle and ANA-12 (0.5 mg/kg body weight) solutions^43^. TDCS was applied 24 h before the 1^st^ day of either NOR or MWM test (Fig. 8a).

Mice subjected to the NOR test were further distributed into ANA-12 and vehicle-injected groups (1% DMSO dissolved in 0.9% NaCl solution). ANA-12 was injected 4 h before and soon after the training day of the NOR test as well as 4 h before the test phase.

Animals subjected to the MWM received ANA-12 injection 4 h before: i) each of the 4 training sessions and ii) the probe test session.

With regard to behavioral tests, given that statistical analysis revealed no significant effect of vehicle administration on the NOR test (P = 1 *vs.* naïve, unpaired Student’s *t*-test), we enrolled in the MWM test only two groups of animals, including tDCS-mice and sham-stimulated mice all injected with curcumin or ANA-12.

### ELISA measurements

Brain tissues were obtained as described for Western blot and stored at −80 °C. Prior to analysis, samples were thawed and then weighed. Lysis buffer (100 mM PIPES pH 7, 500 mM NaCl, 0.2% Triton X-100, 2% BSA, 2 mM EDTA, 200 μM PMSF, 1 × protease, phosphatase-1, and phosphatase-2 inhibitor cocktails from Sigma) was then pipetted into each tube (100 μl per mg of tissue for each left hippocampus). Samples were homogenized, sonicated and centrifuged for 30 min at 16,000 × g at 4 °C. Supernatants were then removed and frozen at −80 °C until analysis. The concentration of Bdnf was determined using the E-Max ImmunoAssay system (Promega) according to the manufacturer’s instructions.

### Statistical analysis

Sample sizes were chosen with adequate statistical power (0.8) according to results of prior pilot data sets or studies, including our own, that used similar methods or paradigms. Sample estimation and statistical analysis were performed using the SigmaPlot 12 software. Data were first tested for equal variance and normality (Shapiro-Wilk test) and then the appropriate statistical tests were chosen. The statistical tests used (i.e., Student’s *t*-test, Mann-Whitney test, one-way ANOVA, two-way ANOVA, two-way RM ANOVA) are indicated in the main text and in the corresponding figure legends for each experiment. *Post-hoc* multiple comparisons were performed with Bonferroni correction.

All statistical tests were two-tailed and the level of significance was set at 0.05. Results are presented as mean ± s.e.m. In the graphs, y-axis error bars represent s.e.m. Analyses were performed in blind.

Criteria of animal exclusion/inclusion were pre-established according to Ethics Committee guidelines but no data were excluded from analysis.

## Additional Information

**How to cite this article**: Podda, M. V. *et al.* Anodal transcranial direct current stimulation boosts synaptic plasticity and memory in mice via epigenetic regulation of Bdnf expression. *Sci. Rep.* **6**, 22180; doi: 10.1038/srep22180 (2016).

## Supplementary Material

Supplementary Information

## References

[b1] Di Lazzaro V. *et al.* Motor cortex plasticity predicts recovery in acute stroke. Cereb. Cortex 20, 1523–1528 (2010). 19805417 10.1093/cercor/bhp216

[b2] Stuchlik A. Dynamic learning and memory, synaptic plasticity and neurogenesis: an update. Front. Behav. Neurosci. 8, 106, doi: 10.3389/fnbeh.2014.00106 (2014). 24744707 PMC3978286

[b3] Marsden W. N. Synaptic plasticity in depression: molecular, cellular and functional correlates. Prog. Neuropsychopharmacol. Biol. Psychiatry 43, 168–184 (2013). 23268191 10.1016/j.pnpbp.2012.12.012

[b4] Rossini P. M., Ferilli M. A., Rossini L. & Ferreri F. Clinical neurophysiology of brain plasticity in aging brain. Curr. Pharm. Des. 19, 6426–6439 (2013). 23432716 10.2174/1381612811319360004

[b5] Brasil-Neto J. P. Learning, memory and transcranial direct current stimulation. Front. Psychiatry 3, 3–80 (2012). 22969734 10.3389/fpsyt.2012.00080 PMC3432476

[b6] Nardone R. Effect of transcranial brain stimulation for the treatment of Alzheimer disease: a review. Int. J. Alzheimers Dis. 2012, 687909, doi: 10.1155/2012/687909 (2012). 22114748 PMC3202129

[b7] Flöel A. tDCS-enhanced motor and cognitive function in neurological diseases. Neuroimage 85, 934–947 (2014). 23727025 10.1016/j.neuroimage.2013.05.098

[b8] Kuo M. F., Paulus W. & Nitsche M. A. Therapeutic effects of non-invasive brain stimulation with direct currents (tDCS) in neuropsychiatric diseases. Neuroimage 85, 948–960 (2014). 23747962 10.1016/j.neuroimage.2013.05.117

[b9] Chhabra H. *et al.* Transcranial direct current stimulation and neuroplasticity genes: implications for psychiatric disorders. Acta Neuropsychiatr. 16, 1–10 (2015). 25877668 10.1017/neu.2015.20

[b10] Creutzfeld O. D., Fromm G. H. & Kapp H. Influence of transcortical dc-currents on cortical neuronal activity. Exp. Neurol. 5, 436–452 (1962). 13882165 10.1016/0014-4886(62)90056-0

[b11] Joucla S. & Yvert B. The “mirror” estimate: an intuitive predictor of membrane polarization during extracellular stimulation. Biophys. J. 96, 3495–3508 (2009). 19413956 10.1016/j.bpj.2008.12.3961 PMC2711410

[b12] Stagg C. J. & Nitsche M. A. Physiological basis of transcranial direct current stimulation. Neuroscientist 17, 37–53 (2011). 21343407 10.1177/1073858410386614

[b13] Rahman A. *et al.* Cellular effects of acute direct current stimulation: somatic and synaptic terminal effects. J. Physiol. 591, 2563–2578 (2013). 23478132 10.1113/jphysiol.2012.247171 PMC3678043

[b14] Bindman L. J., Lippold O. C. & Redfearn J. W. The action of brief polarizing currents on the cerebral cortex of the rat (1) during current flow and (2) in the production of long-lasting after-effects. J. Physiol. 172, 369–382 (1964). 14199369 10.1113/jphysiol.1964.sp007425 PMC1368854

[b15] Reis J. *et al.* Noninvasive cortical stimulation enhances motor skill acquisition over multiple days through an effect on consolidation. Proc. Natl. Acad. Sci. USA 106, 1590–1595 (2009). 19164589 10.1073/pnas.0805413106 PMC2635787

[b16] Flöel A. *et al.* Non-invasive brain stimulation improves object-location learning in the elderly. Neurobiol. Aging 33, 1682–1689 (2012). 21684040 10.1016/j.neurobiolaging.2011.05.007

[b17] Pilato F. *et al.* Synaptic plasticity in neurodegenerative diseases evaluated and modulated by *in vivo* neurophysiological techniques. Mol. Neurobiol. 46, 563–571 (2012). 22821187 10.1007/s12035-012-8302-9

[b18] Fritsch B. *et al.* Direct current stimulation promotes BDNF-dependent synaptic plasticity: potential implications for motor learning. Neuron 66, 198–204 (2010). 20434997 10.1016/j.neuron.2010.03.035 PMC2864780

[b19] Ranieri F. *et al.* Modulation of LTP at rat hippocampal CA3-CA1 synapses by direct current stimulation. J. Neurophysiol. 107, 1868–1880 (2012). 22236710 10.1152/jn.00319.2011

[b20] Rohan J. G., Carhuatanta K. A., McInturf S. M., Miklasevich M. K. & Jankord R. Modulating Hippocampal Plasticity with *In Vivo* Brain Stimulation. J. Neurosci. 35, 12824–12832 (2015). 26377469 10.1523/JNEUROSCI.2376-15.2015 PMC4643097

[b21] Mu J. S., Li W. P., Yao Z. B. & Zhou X. F. Deprivation of endogenous brain-derived neurotrophic factor results in impairment of spatial learning and memory in adult rats. Brain Res. 835, 259–265 (1999). 10415381 10.1016/s0006-8993(99)01592-9

[b22] Waterhouse E. G. & Xu B. New insights into the role of brain-derived neurotrophic factor in synaptic plasticity. Mol. Cell. Neurosci. 42, 81–89 (2009). 19577647 10.1016/j.mcn.2009.06.009 PMC2748315

[b23] Aid T., Kazantseva A., Piirsoo M., Palm K. & Timmusk T. Mouse and rat BDNF gene structure and expression revisited. J. Neurosci. Res. 85, 525–535 (2007). 17149751 10.1002/jnr.21139 PMC1878509

[b24] Pruunsild P., Sepp M., Orav E., Koppel I. & Timmusk T. Identification of cis-elements and transcription factors regulating neuronal activity-dependent transcription of human BDNF gene. J. Neurosci. 31, 3295–3308 (2011). 21368041 10.1523/JNEUROSCI.4540-10.2011 PMC6623925

[b25] Bredy T. W. *et al.* Histone modifications around individual BDNF gene promoters in prefrontal cortex are associated with extinction of conditioned fear. Learn. Mem. 14, 268–276 (2007). 17522015 10.1101/lm.500907 PMC2216532

[b26] Franklin K. B. J. & Paxinos G. T. The Mouse Brain In Stereotaxic Coordinates. Academic Press, New York (1997).

[b27] Liebetanz D. *et al.* Safety limits of cathodal transcranial direct current stimulation in rats. Clin. Neurophysiol. 120, 1161–1167 (2009). 19403329 10.1016/j.clinph.2009.01.022

[b28] Ripoli C. *et al.* Intracellular accumulation of amyloid-β (Aβ) protein plays a major role in Aβ-induced alterations of glutamatergic synaptic transmission and plasticity. J. Neurosci. 34, 12893–12903 (2014). 25232124 10.1523/JNEUROSCI.1201-14.2014 PMC6705320

[b29] Kauer J. A. & Malenka R. C. Synaptic plasticity and addiction. Nat. Rev. Neurosci. 8, 844–858 (2007). 17948030 10.1038/nrn2234

[b30] Curcio L. *et al.* Reduced D-serine levels in the nucleus accumbens of cocaine-treated rats hinder the induction of NMDA receptor-dependent synaptic plasticity. Brain 136, 1216–1230 (2013). 23518710 10.1093/brain/awt036

[b31] Bliss T. V. & Collingridge G. L. A synaptic model of memory: long-term potentiation in the hippocampus. Nature 361, 31–39 (1993). 8421494 10.1038/361031a0

[b32] Nabavi S. *et al.* Engineering a memory with LTD and LTP. Nature 511, 348–352 (2014). 24896183 10.1038/nature13294 PMC4210354

[b33] Cortés-Mendoza J., Díaz, de León-Guerrero S., Pedraza-Alva G. & Pérez-Martínez L. Shaping synaptic plasticity: the role of activity-mediated epigenetic regulation on gene transcription. Int. J. Dev. Neurosci. 31, 359–369 (2013). 23665156 10.1016/j.ijdevneu.2013.04.003

[b34] Karpova N. N. Role of BDNF epigenetics in activity-dependent neuronal plasticity. Neuropharmacology 76, 709–718 (2014). 23587647 10.1016/j.neuropharm.2013.04.002

[b35] Hara D. *et al.* Persistent BDNF exon I-IX mRNA expression following the withdrawal of neuronal activity in neurons. Biochem. Biophys. Res. Commun. 390, 648–653 (2009). 19818730 10.1016/j.bbrc.2009.10.021

[b36] Tian F. *et al.* Dynamic chromatin remodeling events in hippocampal neurons are associated with NMDA receptor mediated activation of Bdnf gene promoter 1. J. Neurochem. 109, 1375–1388 (2009). 19476549 10.1111/j.1471-4159.2009.06058.x

[b37] Caccamo A., Maldonado M. A., Bokov A. F., Majumder S. & Oddo S. CBP gene transfer increases BDNF levels and ameliorates learning and memory deficits in a mouse model of Alzheimer’s disease. Proc. Natl. Acad. Sci. USA 107, 22687–22692 (2010). 21149712 10.1073/pnas.1012851108 PMC3012497

[b38] Pelletier S. J. & Cicchetti F. Cellular and molecular mechanisms of action of transcranial direct current stimulation: evidence from *in vitro* and *in vivo* models. Int. J. Neuropsycopharmacol. 18, 2, doi: 10.1093/ijnp/pyu047 (2015). PMC4368894 25522391

[b39] Kornhauser J. M. *et al.* CREB transcriptional activity in neurons is regulated by multiple, calcium-specific phosphorylation events. Neuron 34, 221–233 (2002). 11970864 10.1016/s0896-6273(02)00655-4

[b40] Liang D. Y., Li X. & Clark J. D. Epigenetic regulation of opioid-induced hyperalgesia, dependence, and tolerance in mice. J. Pain 14, 36–47 (2013). 23273833 10.1016/j.jpain.2012.10.005 PMC3539745

[b41] Zhu X. *et al.* Curcumin alleviates neuropathic pain by inhibiting p300/CBP histone acetyltransferase activity-regulated expression of BDNF and cox-2 in a rat model. PLoS One 9, e91303, doi: 10.1371/journal.pone.0091303 (2014). 24603592 PMC3946321

[b42] Levenson J. M. Regulation of histone acetylation during memory formation in the hippocampus. J. Biol. Chem. 279, 40545–40559 (2004). 15273246 10.1074/jbc.M402229200

[b43] Cazorla M. *et al.* Identification of a low-molecular weight TrkB antagonist with anxiolytic and antidepressant activity in mice. J. Clin. Invest. 121, 1846–1857 (2011). 21505263 10.1172/JCI43992 PMC3083767

[b44] Peineau S. *et al.* The role of GSK-3 in synaptic plasticity. Br. J. Pharmacol. 153, S428–S437 (2008). 18311157 10.1038/bjp.2008.2 PMC2268071

[b45] Ji Y. *et al.* Acute and gradual increases in BDNF concentration elicit distinct signaling and functions in neurons. Nat. Neurosci. 13, 302–309 (2010). 20173744 10.1038/nn.2505 PMC4780419

[b46] Liebetanz D. *et al.* After-effects of transcranial direct current stimulation (tDCS) on cortical spreading depression. Neurosci. Lett. 398, 85–90 (2006). 16448754 10.1016/j.neulet.2005.12.058

[b47] Cambiaghi M. *et al.* Brain transcranial direct current stimulation modulates motor excitability in mice. Eur. J. Neurosci. 31, 704–709 (2010). 20141528 10.1111/j.1460-9568.2010.07092.x

[b48] Pedron S., Monnin J., Haffen E., Sechter D. & Van Waes V. Repeated transcranial direct current stimulation prevents abnormal behaviors associated with abstinence from chronic nicotine consumption. Neuropsychopharmacol. 39, 981–988 (2014). 10.1038/npp.2013.298 PMC3924532 24154668

[b49] Kamida T. *et al.* Transcranial direct current stimulation decreases convulsions and spatial memory deficits following pilocarpine-induced status epilepticus in immature rats. Behav. Brain Res. 217, 99–103 (2011). 20826186 10.1016/j.bbr.2010.08.050

[b50] Bolzoni F., Bączyk M. & Jankowska E. Subcortical effects of transcranial direct current stimulation in the rat. J. Physiol. 591, 4027–4042 (2013). 23774279 10.1113/jphysiol.2013.257063 PMC3764643

[b51] Vorhees C. V. & Williams M. T. Assessing spatial learning and memory in rodents. ILAR J. 55, 310–332 (2014). 25225309 10.1093/ilar/ilu013 PMC4240437

[b52] Cohen S. J. & Stackman R. W. Jr. Assessing rodent hippocampal involvement in the novel object recognition task. A review. Behav. Brain Res. 285, 105–117 (2015). 25169255 10.1016/j.bbr.2014.08.002 PMC7008635

[b53] Lang N. *et al.* Preconditioning with transcranial direct current stimulation sensitizes the motor cortex to rapid-rate transcranial magnetic stimulation and controls the direction of after-effects. Biol. Psychiatry 56, 634–639 (2004). 15522246 10.1016/j.biopsych.2004.07.017

[b54] Siebner H. R. *et al.* Preconditioning of low-frequency repetitive transcranial magnetic stimulation with transcranial direct current stimulation: evidence for homeostatic plasticity in the human motor cortex. J. Neurosci. 24, 3379–3385 (2004). 15056717 10.1523/JNEUROSCI.5316-03.2004 PMC6730024

[b55] Nitsche M. A. *et al.* Timing-dependent modulation of associative plasticity by general network excitability in the human motor cortex. J. Neurosci. 27, 3807–3812 (2007). 17409245 10.1523/JNEUROSCI.5348-06.2007 PMC6672399

[b56] Clark V. P. *et al.* TDCS guided using fMRI significantly accelerates learning to identify concealed objects. Neuroimage 59, 117–128 (2012). 21094258 10.1016/j.neuroimage.2010.11.036 PMC3387543

[b57] Cheeran B. *et al.* A common polymorphism in the brain-derived neurotrophic factor gene (BDNF) modulates human cortical plasticity and the response to rTMS. J. Physiol. 586, 5717–5725 (2008). 18845611 10.1113/jphysiol.2008.159905 PMC2655403

[b58] Chaieb L., Antal A., Ambrus G. G. & Paulus W. Brain-derived neurotrophic factor: its impact upon neuroplasticity and neuroplasticity inducing transcranial brain stimulation protocols. Neurogenetics 15, 1–11 (2014). 24567226 10.1007/s10048-014-0393-1

[b59] Alarcòn J. M. *et al.* Chromatin acetylation memory and LTP are impaired in CBP +/− mice: a model for the cognitive deficit in Rubinstein-Taybi syndrome and its amelioration. Neuron 42, 947–959 (2004). 15207239 10.1016/j.neuron.2004.05.021

[b60] Levenson J. M. & Sweatt J. D. Epigenetic mechanisms in memory formation. Nat. Rev. Neurosci. 6, 108–118 (2005). 15654323 10.1038/nrn1604

[b61] Tsankova N. M. *et al.* Sustained hippocampal chromatin regulation in a mouse model of depression and antidepressant action. Nat. Neurosci. 9, 519–525 (2006). 16501568 10.1038/nn1659

[b62] Kailainathan S. *et al.* Activation of a synapse weakening pathway by human Val66 but not Met66 pro-brain-derived neurotrophic factor (proBDNF). Pharmacol. Res. 104, 97–107 (2016). 26687096 10.1016/j.phrs.2015.12.008 PMC4773404

[b63] Tabuchi A., Sakaya H., Kisukeda T., Fushiki H. & Tsuda M. Involvement of an upstream stimulatory factor as well as cAMP-responsive element-binding protein in the activation of brain-derived neurotrophic factor gene promoter I. J. Biol. Chem. 277, 35920–35931 (2002). 12114522 10.1074/jbc.M204784200

[b64] Grassi C., D’Ascenzo M., Valente A. & Battista Azzena G. Ca^2+^ channel inhibition induced by nitric oxide in rat insulinoma RINm5F cells. Pflugers Arch. 437, 241–247 (1999). 9929565 10.1007/s004240050775

[b65] Shipton O. A. *et al.* Left-right dissociation of hippocampal memory processes in mice. Proc. Natl. Acad. Sci. USA 111, 15238–15243 (2014). 25246561 10.1073/pnas.1405648111 PMC4210314

[b66] Attar A. *et al.* Protection of primary neurons and mouse brain from Alzheimer’s pathology by molecular tweezers. Brain 135, 3735–3748 (2012). 23183235 10.1093/brain/aws289 PMC3525056

[b67] Chegaev K. *et al.* NO-donor thiacarbocyanines as multifunctional agents for Alzheimer’s disease. Bioorg. Med. Chem. 23, 4688–4698 (2015). 26078011 10.1016/j.bmc.2015.05.050

[b68] Caria M. A., Melis F., Podda M. V., Solinas A. & Deriu F. Does long-term potentiation occur in guinea-pig Deiters’ nucleus? Neuroreport 7, 2303–2307 (1996). 8951843 10.1097/00001756-199610020-00007

[b69] Leone L. *et al.* Epigenetic modulation of adult hippocampal neurogenesis by extremely low-frequency electromagnetic fields. Mol. Neurobiol. 49, 1472–1486 (2014). 24532268 10.1007/s12035-014-8650-8

[b70] Podda M. V. *et al.* Extremely low-frequency electromagnetic fields enhance the survival of newborn neurons in the mouse hippocampus. Eur. J. Neurosci. 39, 893–903 (2014). 24382162 10.1111/ejn.12465

