# Supplementary material for: Anodal transcranial direct current stimulation boosts synaptic plasticity and memory in mice via epigenetic regulation of Bdnf expression
Source: Sci Rep. 2016 Feb 24;6:22180. doi: 10.1038/srep22180 (PMC4764914; doi:10.1038/srep22180)
Supplement: Supplementary Information [file srep22180-s1.doc]

**Supplementary Information**

Supplementary Figures 1-8 and Supplementary Tables 1-5

**Anodal transcranial direct current stimulation boosts synaptic plasticity and memory in mice via epigenetic regulation of Bdnf expression**

Maria Vittoria Podda, Sara Cocco, Alessia Mastrodonato, Salvatore Fusco, Lucia Leone, Saviana Antonella Barbati, Claudia Colussi, Cristian Ripoli, and Claudio Grassi

**Supplementary Figures**


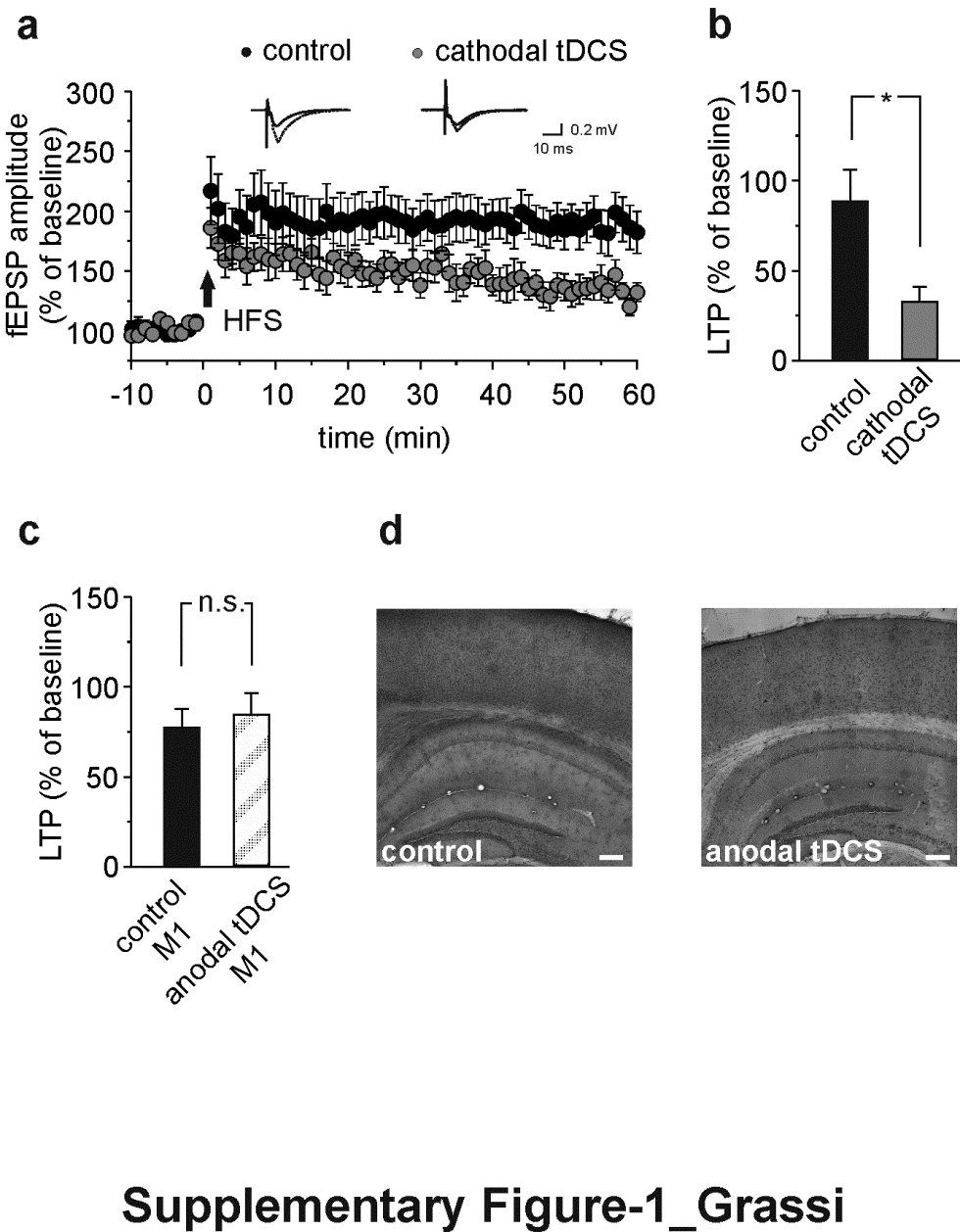


**Supplementary Figure 1.** (**a**) Time course of LTP at CA3-CA1 synapses induced by HFS delivered at time 0 (arrow) in hippocampal slices obtained from control (n=9 slices from 4 mice) and cathodal tDCS-mice (n=10 slices from 4 mice). (**b**) Bar graphs showing LTP values observed in control and cathodal tDCS-mice (P=0.004, unpaired Student’s *t*-test). (**c**) Bar graphs comparing the magnitude of LTP elicited in hippocampal slices from control and tDCS-mice stimulated over the left primary motor cortex (P=0.59 *vs.* control, unpaired Student’s *t*-test; n=6 slices from 2 tDCS-mice and n=8 slices from 2 control mice). (**d**) Representative hippocampal slices containing the left hippocampus stained with haematoxylin and eosin obtained from control and anodal tDCS-mice (n=3 mice for each group). Scale bars: 200 μm. Data are expressed as mean ± s.e.m. *P<0.05; n.s., not significant *vs.* control mice.


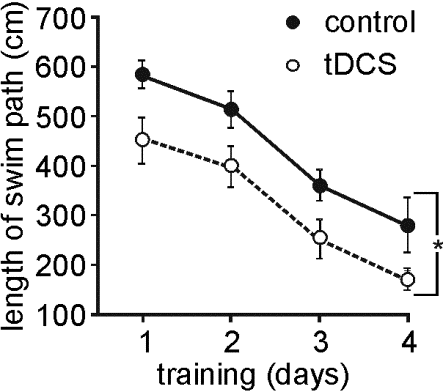


**Supplementary Figure 2.** Length of swim path to the hidden platform in control and tDCS-mice 24 h after stimulation. In the acquisition session of the MWM all mice successfully acquired the task with the length of the swim path decreasing across training days (main effect of days: F_3,48_=31.7, P<0.001; two-way RM ANOVA). Notably tDCS-mice performed better than control mice (n=9 mice/group; main effect of treatment: F_1,48_=14.1, P=0.002; two-way RM ANOVA). As observed for latency, tDCS-mice showed reduced length of the swim path already at day 1 (main effect of tDCS: F_1,80_=6.8, P=0.019; two-way RM ANOVA). No differences were found between the two groups of mice in the first two trials (P=0.67 and P=0.41 for trial 1 and trial 2, respectively; two-way RM ANOVA followed by Bonferroni *post-hoc* test). Overall no differences in the average swim speed could be detected at all time points between tDCS-mice and control group (main effect of tDCS: F_1,48_=0.001, P=0.97; two-way RM ANOVA). Data are expressed as mean ± s.e.m. *P<0.05.


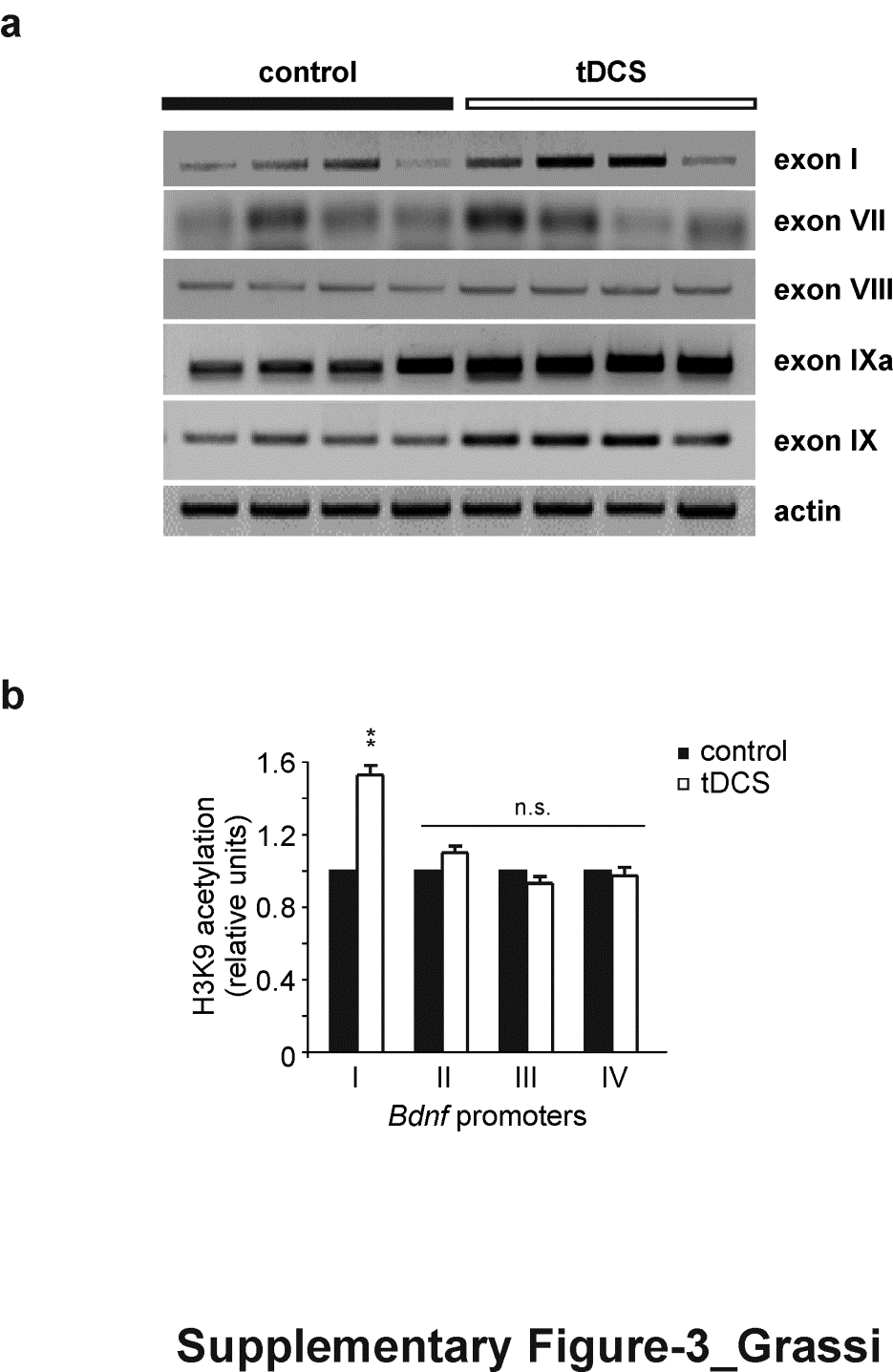


**Supplementary Figure 3. (a)** Semiquantitative RT-PCR analysis of hippocampal extracts from 4 control and 4 tDCS-mice showing increased expression of *Bdnf* mRNAs of exons I, VII, VIII, IXa and IX 24 h after stimulation protocol. (**b**) ChIP assays performed on hippocampi from mice sacrificed 24 h after sham stimulation (n=3 mice) or anodal tDCS (n=3 mice) revealed that anodal tDCS significantly enhanced H3K9 acetylation at promoter I (P=4E-7) but it did not affect the level of acetylation at promoters II, III, IV. Data are expressed as mean ± s.e.m. **P<0.001; n.s., not significant *vs.* control mice.

**
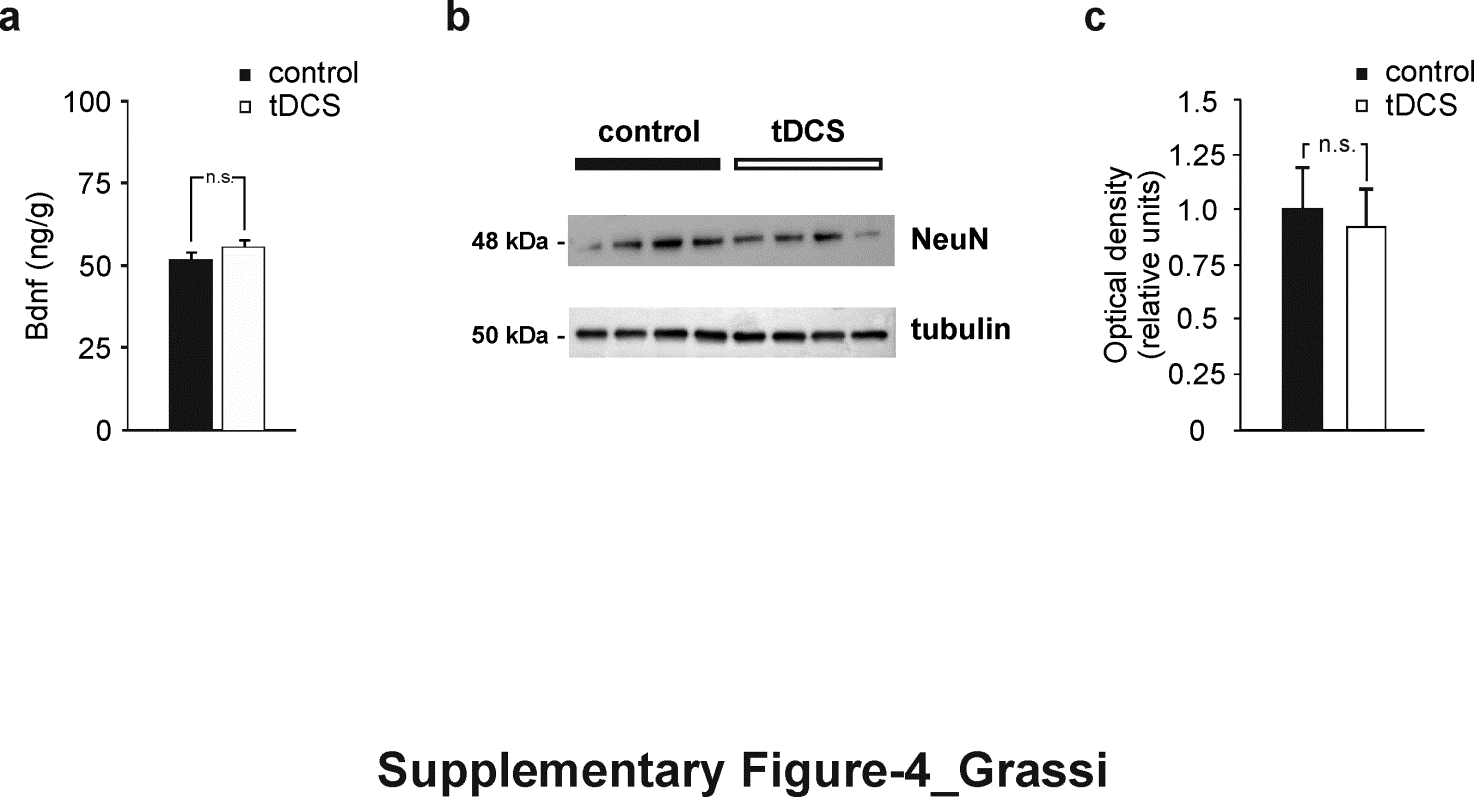
**

**Supplementary Figure 4. (a)** Results from ELISA showing that 24 hours after tDCS Bdnf levels in the cerebellum were similar in control (n=4) and tDCS-mice (n=6; P=0.19, unpaired Student’s *t*-test). The assay was performed in duplicate. (**b**) Western blot analysis revealing that expression of the neuronal protein NeuN was not affected by tDCS. (**c**) Band densitometry normalized to tubulin (P=0.73 vs control; n= 4 tDCS-mice *vs.* n=4 controls; unpaired Student’s *t*-test). Data are expressed as mean ± s.e.m.; n.s., not significant *vs.* control.

**
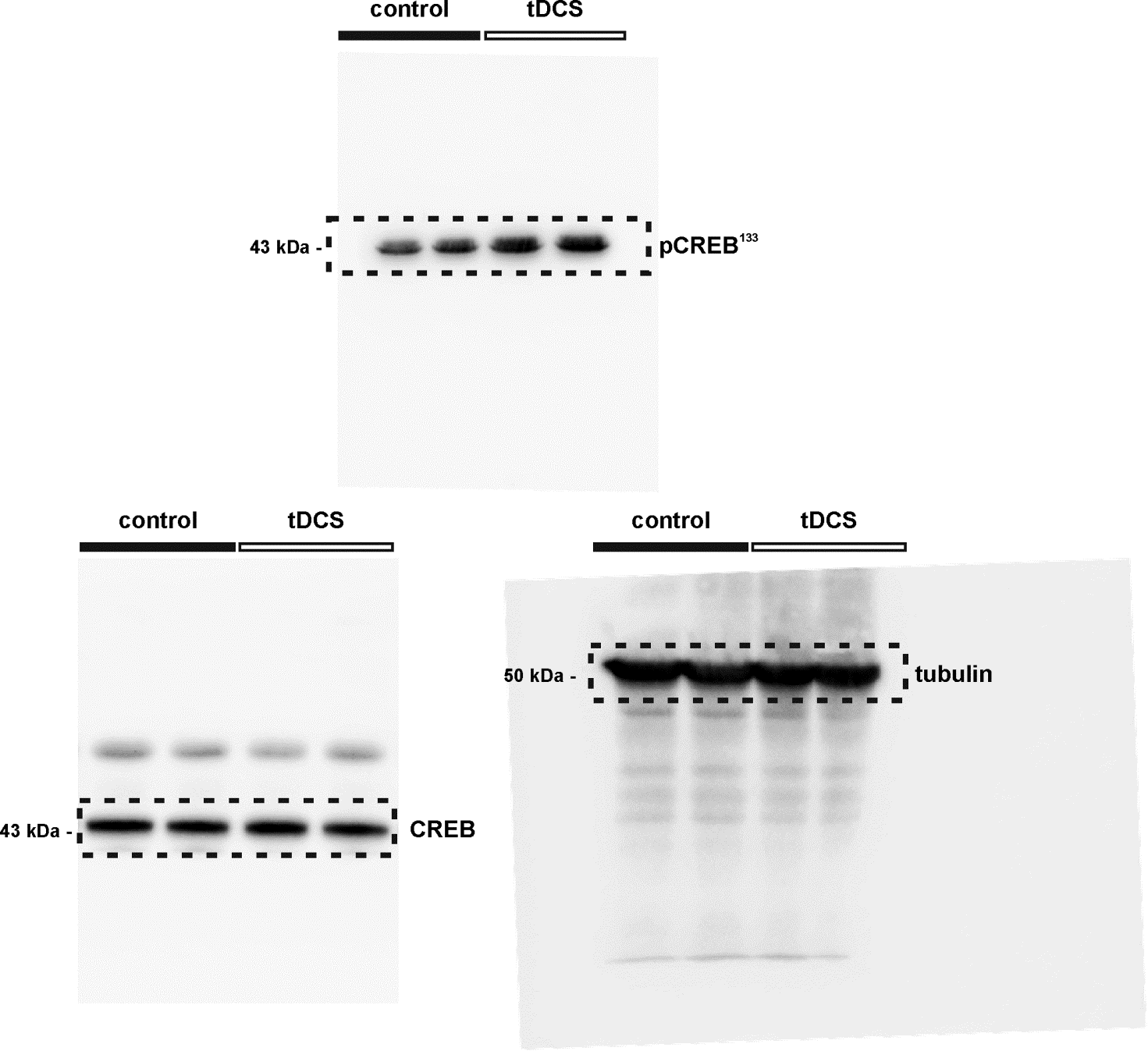
**

**Supplementary Figure 5.** Full uncropped scans of pCREB^133^, CREB, tubulin Western blots shown in Fig. 6a (dashed boxes).

**
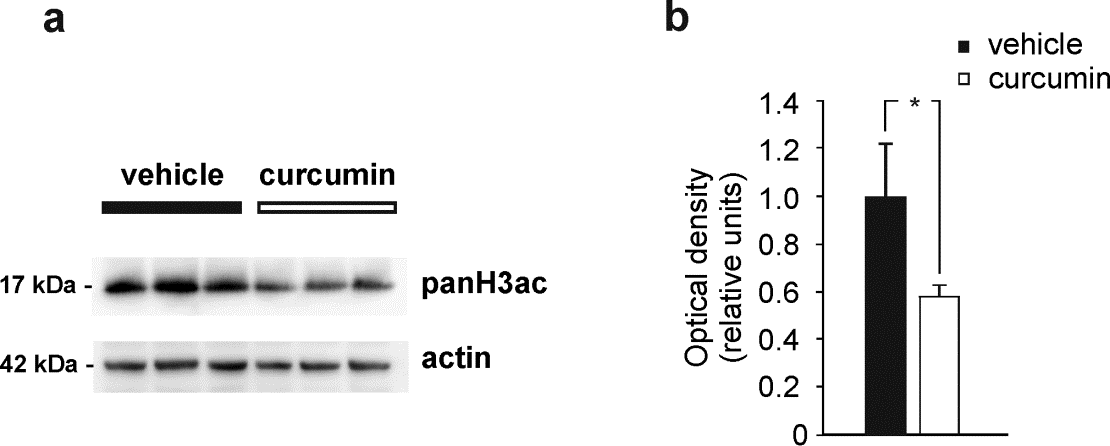
**

**Supplementary Figure 6.** (**a**) Western blot analysis of hippocampal homogenates revealing that histone H3 acetylation, as detected by pan-acetyl histone H3 antibody (panH3ac), was significantly decreased by curcumin treatment (50 mg/kg body weight, i.p., for 3 consecutive days; -42% *vs.* vehicle; P=0.008, unpaired Student’s *t*-test; n=3 for each group). (**b**) Band densitometry normalized to actin. Experiments were performed in duplicate. Optical density values are expressed as fold-changes *vs.*vehicle taken as 1. Data are expressed as mean ± s.e.m. *P<0.05.


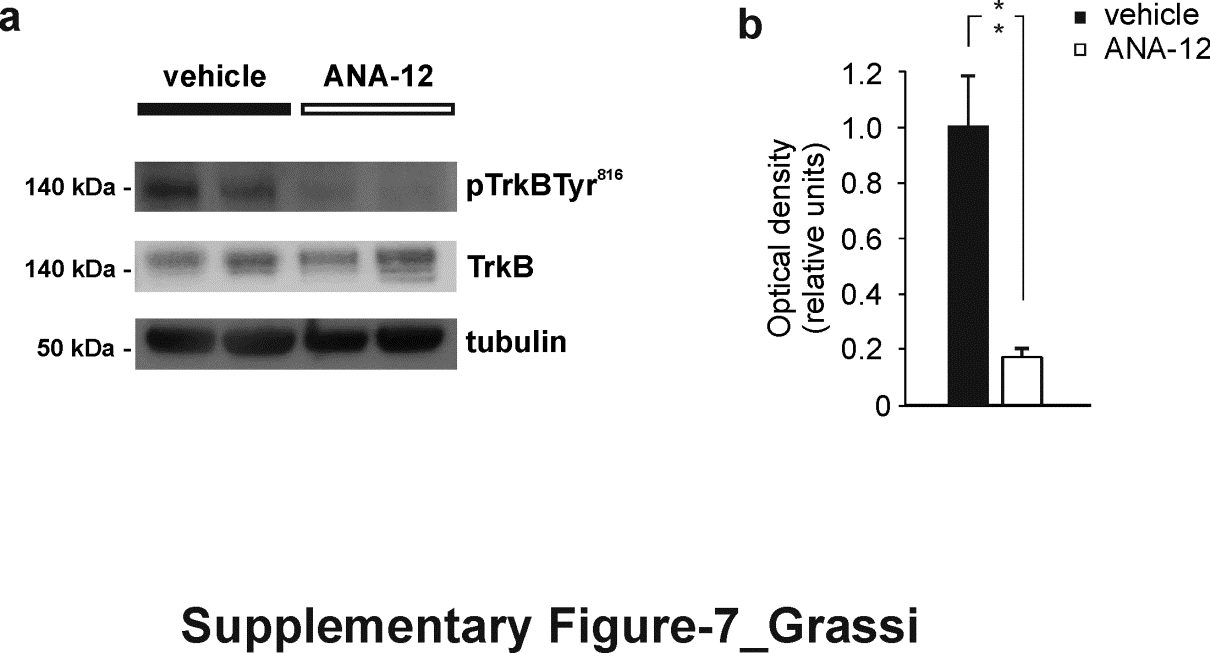


**Supplementary Figure 7.** (**a**) Western blot analysis of hippocampal homogenates revealing that the levels of TrkB phosphorylated at Tyrosine 816 were decreased in mice treated with ANA-12 (0.5 mg/kg body weight, i.p., for 3 consecutive days) compared to vehicle-injected mice. Experiments were performed in duplicate. (**b**) Band densitometry of pTrkBTyr^816^/TrkB ratio normalized to tubulin (P=0.0006; unpaired Student’s *t*-test; n=5 ANA-12 injected mice *vs.* n=4 vehicle-injected mice).


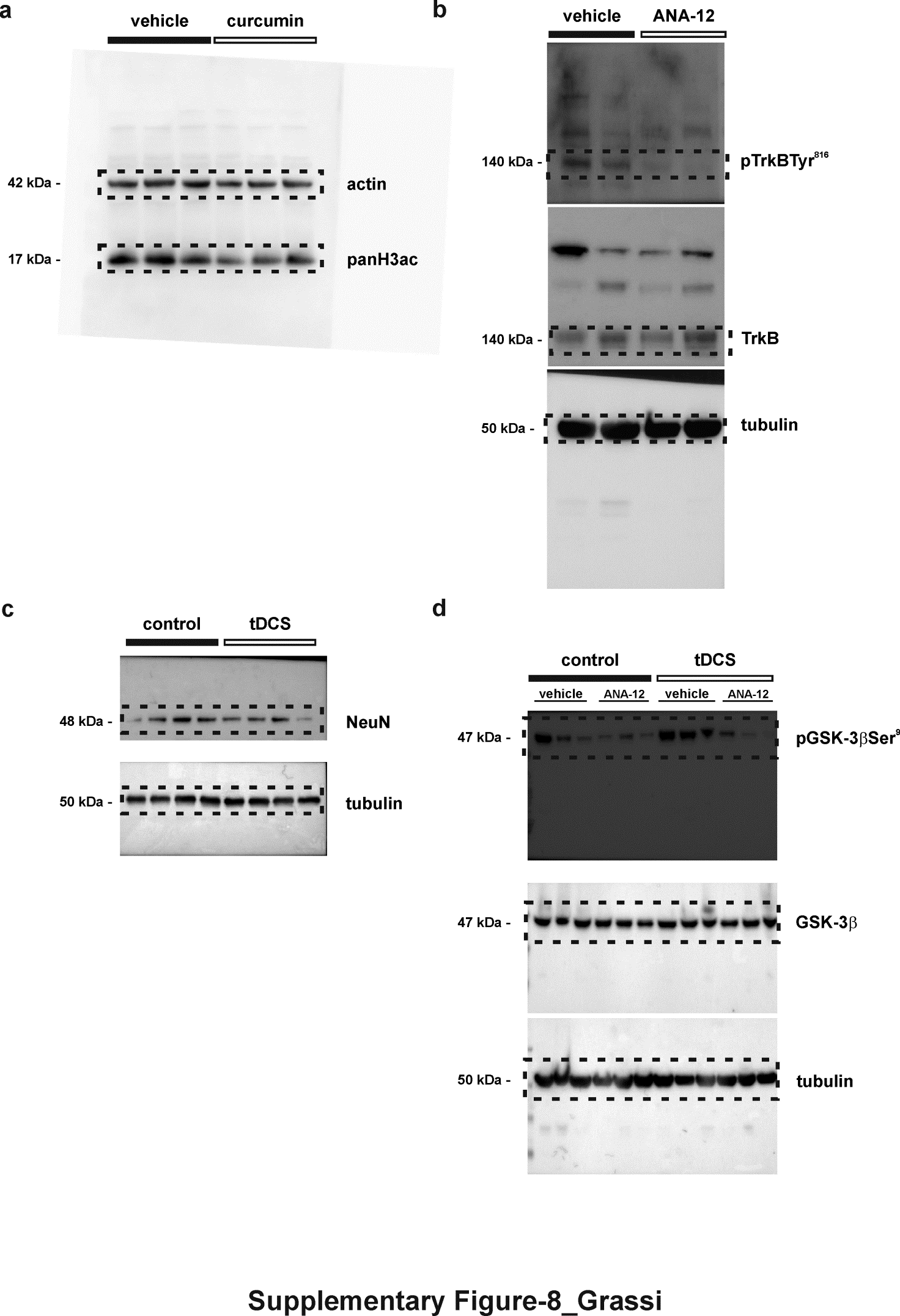


**Supplementary Figure 8.** Full uncropped scans of (**a**) panH3ac, (**b**) pTrKB, (**c**), NeuN, (**d**) pGSK-3β Western blots shown in Supplementary Fig. 6, Supplementary Fig. 7, Supplementary Fig. 4 and Figure 8, respectively (dashed boxes).

**Supplementary Tables**

**Supplementary Table 1.** Statistical analysis of LTP.

| #Slices | P values from paired Student’s *t*-test comparing fEPSP amplitudes 5 min before HFS  *vs.* fEPSP amplitudes 55-60 min after HFS in all LTP experimental sets | | | | | | | | | | | |
| --- | --- | --- | --- | --- | --- | --- | --- | --- | --- | --- | --- | --- |
|  | LTP 2h after atDCS (Fig. 2a,b) | | LTP 1 week after atDCS (Fig. 4a,b) | | LTP curcumin 1 week after atDCS  (Fig. 7e,f) | | | | LTP 2h after ctDCS (Suppl. Fig. 1a,b) | | LTP 2h after M1 atDCS (Suppl. Fig. 1c) | |
|  | sham | atDCS | sham | atDCS | Vehicle  sham | Vehicle  atDCS | Curcumin  sham | Curcumin  atDCS | sham | ctDCS | sham | M1  atDCS |
| Slice 1 | 7E-18 | 4E-15 | 1E-11 | 1E-17 | 2E-09 | 4E-13 | 2E-15 | 2E-13 | 2E-20 | 2E-16 | 4E-17 | 4E-21 |
| Slice 2 | 4E-18 | 4E-20 | 1E-17 | 1E-18 | 2E-14 | 4E-12 | 6E-20 | 2E-10 | 5E-10 | 4E-01 | 8E-17 | 1E-14 |
| Slice 3 | 3E-15 | 2E-19 | 5E-15 | 2E-21 | 1E-10 | 2E-12 | 2E-14 | 2E-15 | 2E-21 | 9E-04 | 2E-11 | 3E-17 |
| Slice 4 | 2E-17 | 3E-23 | 1E-18 | 6E-21 | 2E-12 | 6E-16 | 6E-20 | 4E-13 | 5E-12 | 3E-08 | 1E-13 | 2E-12 |
| Slice 5 | 1E-16 | 1E-14 | 3E-18 | 1E-17 | 6E-19 | 5E-16 | 6E-16 | 1E-13 | 2E-16 | 4E-12 | 5E-20 | 1E-16 |
| Slice 6 | 2E-14 | 2E-20 | 2E-17 | 7E-18 | 2E-07 | 2E-15 | 3E-13 | 1E-06 | 1E-10 | 8E-04 | 4E-13 | 6E-15 |
| Slice 7 | 6E-18 | 3E-16 | 5E-13 | 8E-14 | 4E-16 | 7E-18 | 5E-11 | 5E-07 | 7E-14 | 1E-06 | 8E-16 |  |
| Slice 8 | 6E-16 | 3E-17 | 4E-13 | 2E-12 | 2E-18 | 3E-16 | 8E-09 | 5E-11 | 1E-04 | 8E-03 | 8E-13 |  |
| Slice 9 | 4E-16 | 7E-18 | 2E-19 | 5E-13 | 2E-12 | 8E-12 | 1E-06 | 2E-13 | 3E-09 | 1E-05 |  |  |
| Slice10 | 1E-15 | 5E-21 |  | 5E-13 | 2E-09 |  |  |  |  | 9E-03 |  |  |
| Slice11 | 9E-19 |  |  | 7E-19 | 6E-11 |  |  |  |  |  |  |  |
| Slice12 | 4E-08 |  |  |  | 9E-15 |  |  |  |  |  |  |  |
| Slice13 | 4E-11 |  |  |  |  |  |  |  |  |  |  |  |
| Slice14 | 4E-25 |  |  |  |  |  |  |  |  |  |  |  |
| Slice15 | 1E-08 |  |  |  |  |  |  |  |  |  |  |  |
| Slice16 | 2E-14 |  |  |  |  |  |  |  |  |  |  |  |

Abbreviations: #slices, number of slices tested in each LTP experimental set; atDCS, anodal transcranial direct current stimulation; ctDCS, cathodal transcranial direct current stimulation; M1, primary motor cortex

**Supplementary Table 2. Primer sequences used for ChIP analysis in this study.**

| Gene | Primer sequence | |
| --- | --- | --- |
| *Bdnf* PI | FW | 5’-ACGAACTTTTCTAAGAAGTTTCC-3’ |
|  | RV | 5’-CTGCACCAGCCGGCTACTGC-3’ |
| Abbreviations: *Bdnf*, brain-derived neurotrophic factor; PI, promoter I; FW, forward; RV, reverse | | |

**Supplementary Table 3.** Primer sequences used for Semiquantitative PCR analysis in this study.

| Gene | Primer sequence | | T_m_ (°C) |
| --- | --- | --- | --- |
| RT *Bdnf* Exon I | FW | 5’-GACCTGAGCAGTGGGCAAAGG-3’ | 60 |
|  | RV | 5’-GAAGTGTACAAGTCCGCGTCC-3’ |  |
| RT *Bdnf* Exon II | FW | 5’-AAGTGGAAGAAACCGTCTAGAGC-3’ | 60 |
|  | RV | 5’-GAAGTGTACAAGTCCGCGTCC-3’ |  |
| RT *Bdnf* Exon III | FW | 5’-TTCTATCATCCCTCCCCGAGAG-3’ | 60 |
|  | RV | 5’-GAAGTGTACAAGTCCGCGTCC-3’ |  |
| RT *Bdnf* Exon IV | FW | 5’-CTGCCTAGATCAAATGGAGCTTC-3’ | 60 |
|  | RV | 5’-GAAGTGTACAAGTCCGCGTCC-3’ |  |
| RT *Bdnf* Exon V | FW | 5’-CTCTGTGTAGTTTCATTGTGTGTTC-3’ | 60 |
|  | RV | 5’-GAAGTGTACAAGTCCGCGTCC-3’ |  |
| RT *Bdnf* Exon VI | FW | 5’-GCTGGCTGTCGCACGGTTCC-3’ | 60 |
|  | RV | 5’-GAAGTGTACAAGTCCGCGTCC-3’ |  |
| RT *Bdnf* Exon VII | FW | 5’-CCTGAAAGGGTCTGCGGAACTC-3’ | 60 |
|  | RV | 5’-GAAGTGTACAAGTCCGCGTCC-3’ |  |
| RT *Bdnf* Exon VIII | FW | 5’-GTGTCGCTGCGCCTCAGTGG-3’ | 60 |
|  | RV | 5’-GAAGTGTACAAGTCCGCGTCC-3’ |  |
| RT *Bdnf* Exon IXa | FW | 5’-CAAAGCTGCTAAAGCGGGAGG-3’ | 60 |
|  | RV | 5’-GAAGTGTACAAGTCCGCGTCC-3’ |  |
| RT *Bdnf* Exon IX | FW | 5’-TGGCTGACACTTTTGAGCACG-3’ | 60 |
|  | RV | 5’-GAAGTGTACAAGTCCGCGTCC-3’ |  |
| *Bdnf* PI | FW | 5’-TCTTCGATTCACGCAGTTGTTCC-3’ | 59 |
|  | RV | 5’-CTGCACCAGCCGGCTACTGC-3’ |  |
| *Bdnf* PII | FW | 5’-CCGTCTTGTATTCCATCCTTTGC-3’ | 59 |
|  | RV | 5’-CAACTCCACCACTATCCTCACC-3’ |  |
| *Bdnf* PIII | FW | 5’-GTGAGAACCTGGGGCAAATCG-3’ | 59 |
|  | RV | 5’-GCACACACACCACACACAAGC-3’ |  |
| *Bdnf* PIV | FW | 5’-CATGCAATGCCCTGGAACGG-3’ | 59 |
|  | RV | 5’-GTGGAAATTGCATGGCGGAGG-3’ |  |
| Abbreviations: RT, reverse-transcriptase; *Bdnf*, brain-derived neurotrophic factor; PI, promoter I; PII, promoter II; PIII, promoter III; PIV, promoter IV; FW, forward; RV, reverse; T_m_, melting temperature | | | |

**Supplementary Table 4.** Primer sequences used for quantitative Real-Time PCR analysis in this study.

| Gene | Accession n° | Primer sequences | | T_m_ (°C) |
| --- | --- | --- | --- | --- |
| *Bdnf*  Exon I | EF125669.1 | FW | 5’-TGAGAGTTGAAGCTTTGCGG-3’ | 55 |
|  |  | RV | 5’-ATTGTGGCTTTGCTGTCCTG-3’ |  |
| *Bdnf*  Exon IV | EF125685.1 | FW | 5’-AGCATGAAATCTCCCAGCCT-3’ | 55 |
|  |  | RV | 5’-CGGTCCCCAAGGTTCTAGAC-3’ |  |
| *Bdnf*  Exon IX | NM_001048142.1 | FW | 5’-TGGCTGACACTTTTGAGCAC-3’ | 55 |
|  |  | RV | 5’-GTTTGCGGCATCCAGGTAAT-3’ |  |
| Gapdh | NM_008084.2 | FW | 5’-TCCATGACAACTTTGGCATT-3’ | 55 |
|  |  | RV | 5’-GTTGCTGTTGAAGTCGCAGG-3’ |  |

Abbreviations: Gapdh, glyceraldehyde-3-phosphate deydrogenase; FW, forward; RV, reverse; T_m_, melting temperature

**Supplementary Table 5.** Primary and secondary antibodies used in this study.

| Primary Antibody | Host | Catalogue reference | |
| --- | --- | --- | --- |
| pCREB (Ser133) | Rabbit | Cell Signalling | #87G3 |
| pCREB (Ser133) | Rabbit | Millipore | 17-10131 |
| CREB | Mouse | Cell Signalling | #48H2 |
| Tubulin | Mouse | Sigma | T9026 |
| β-actin | Rabbit | Abcam | Ab8227 |
| Acetyl Histone H3K9 | Rabbit | Millipore | 07-352 |
| CBP | Rabbit | Abcam | Ab2832 |
| GSK-3β | Rabbit | Cell Signalling | #12456 |
| pGSK-3β (Ser9) | Rabbit | Cell Signalling | #9336 |
| Phospho-TrkA(Tyr785)/TrkB(Tyr816) | Rabbit | Cell Signalling | #4168 |
| TrkB | Rabbit | Cell Signalling | #4606 |
| PanH3ac | Rabbit | Millipore | 06-599 |
| NeuN | Mouse | Millipore | MAB377 |

| Secondary Antibody | Catalogue reference | |
| --- | --- | --- |
| Anti-mouse | Cell Signalling | #7076 |
| Anti-rabbit | Cell Signalling | #7074 |
